# Neoadjuvant camrelizumab combined with metronomic chemotherapy in patients with advanced esophageal squamous cell carcinoma: a pilot randomized phase 2 trial

**DOI:** 10.1186/s12916-026-04758-3

**Published:** 2026-03-03

**Authors:** Zhiming Chen, Wenshuai Li, Yumeng Guo, Yongjun Zhu, Yang Song, Jiayan Wu, Wanwei Zheng, Yujen Tseng, Lishuang Lin, Feng Tang, Beibei Mao, Peng Zhao, Xiangyu Guo, Shiman Fu, Gang Chen, Ning Wu, Bin Lv, Yao Liu, Shenyang Zhao, Shaocong Mo, Kewei Ma, Kaiyi Fu, Hongyang Zhang, Jun Zhang, Feifei Luo, Zhongguang Luo, Jie Liu

**Affiliations:** 1 Department of Digestive Diseases, Huashan Hospital, Fudan University, 12 Wulumuqi Middle Road, Shanghai, 200040 China; 2 Department of Thoracic Surgery, Huashan Hospital, Fudan University, Shanghai, 200040 China; 3 Department of Pathology, Huashan Hospital, Fudan University, Shanghai, China; 4 Genecast Biotechnology Co., Ltd, Wuxi, Jiangsu China; 5 National Clinical Research Centre for Aging and Medicine, Huashan Hospital, Fudan University, 12 Wulumuqi Middle Road, Shanghai, 200040 China

**Keywords:** Esophageal squamous cell carcinoma (ESCC), Neoadjuvant therapy, Metronomic chemotherapy, Camrelizumab

## Abstract

**Background:**

Compared to conventional chemotherapy, metronomic chemotherapy (MCT), with lower drug dosage which may cause less damage to the immune system, has shown potential for synergy in combination with PD-1-based immunotherapy. However, this synergistic immunotherapy efficacy in neoadjuvant setting for advanced esophageal squamous cell carcinoma (ESCC) requires further clinical validation.

**Methods:**

This pilot phase 2, single-center, randomized clinical trial enrolled 30 untreated patients with resectable stage II or III ESCC. Participants were randomly assigned to either the MCT group (paclitaxel, cisplatin, and 5-fluorouracil) or the IO + MCT group (same regimen plus camrelizumab). Primary outcomes included the pCR rate after neoadjuvant therapy, and the safety of each regimen assessed by adverse events. Digital spatial profiling (DSP-WTA), multiplex immunofluorescent staining (mIF), and bulk RNA sequencing were performed to explore the possible therapeutic mechanisms.

**Results:**

Twenty-four patients (13 in MCT, 11 in IO + MCT) underwent R0 resection. The pCR rates were 15.4% in the MCT group and 54.5% in the IO + MCT group. Both treatments were well tolerated, with manageable side effects. DSP-WTA and mIF revealed that IO + MCT effectively decreased the number of tumor-infiltrating T cells with the positive expression of terminal exhaustion marker CD39 and increased the number of primary and secondary follicle-like tertiary lymphoid structures (TLSs), particularly in pCR patients.

**Conclusions:**

Neoadjuvant MCT combined with camrelizumab led to an increased pCR rate (54.5 vs. 16.7%) in ESCC patients compared to MCT alone. This combination therapy may offer a promising approach for enhancing cancer treatment outcomes.

**Trial registration:**

ClinicalTrials.gov identifier: ChiCTR2000039638.

**Supplementary Information:**

The online version contains supplementary material available at 10.1186/s12916-026-04758-3.

## Background

According to the NCCN guidelines, the recommended first-line treatment for locally advanced resectable esophageal cancer is neoadjuvant chemoradiotherapy followed by surgical intervention [1, 2]. However, suboptimal rates of tumor regression underscore the need for more effective therapeutic strategies. The phase III ESOPEC trial, a recently reported study in 2024 ASCO, compared the efficacy of perioperative chemotherapy (FLOT) with neoadjuvant chemoradiotherapy (CROSS) in patients with resectable locally advanced esophageal and gastroesophageal junction adenocarcinoma [3]. The trial revealed that neoadjuvant chemotherapy could enhance the pathological complete response (pCR) rate to 16.8% [3, 4], notably higher than the 10% rate achieved with neoadjuvant chemoradiotherapy, while also demonstrating a 3-year overall survival (OS) rate of 57%. Concurrently, immune checkpoint inhibitors (ICI) have emerged as a significant force in the treatment of esophageal squamous cell carcinoma (ESCC), with clinical trials focusing on PD-1/PD-L1 inhibitors yielding promising results in both first-line and second-line treatments [4, 5]. These advances have ignited hope for patients with locally advanced resectable ESCC. Moreover, the integration of immunotherapy into neoadjuvant protocols for ESCC has demonstrated improved objective response rates (ORR), higher pCR rates, and enhanced 3-year OS rates. Recent clinical studies on small locally advanced resectable ESCC patient cohorts have indicated that neoadjuvant therapy, primarily involving anti-PD-1/PD-L1 therapy combined with paclitaxel and either carboplatin or cisplatin, exhibited promising results, with R0 resection rates of 80–100%, major pathological remissions (MPR) rates of 30–78%, and pCR rates of 23–33% [6–10]. Moreover, the latest phase III clinical trial of perioperative immunotherapy for esophageal cancer ESCORT-NEO trial has further highlighted the benefits of neoadjuvant immunotherapy, with a significant increase in pCR rates compared to chemotherapy alone, showing an improvement of 15.4–28% versus 4.7% [11].

Despite these promising developments, the combination of chemotherapy and immunotherapy is associated with significant adverse effects, with grade 3–4 adverse events occurring at a rate of up to 34%, leading to poor patient tolerance and even treatment-related adverse effects (TRAEs)-related termination of clinical trials [11]. Additionally, distant relapse rates remain concerning, particularly for those with residual disease post-resection. These factors underscore the imperative to explore innovative, multimodal therapeutic strategies aimed at further enhancing survival benefits and reducing TRAEs for these patients.

The favorable safety profile especially in reduced toxicity and decreased probability of acquired therapeutic resistance make metronomic chemotherapy (MCT) an enticing alternative, as compared to the conventional maximum tolerated dose (MTD) chemotherapy. Meanwhile, previous clinical trials have demonstrated the beneficial efficacy of MCT, which significantly improved the failure-free survival with a manageable safety profile [12]. For instance, in metastatic breast cancer, MCT (oral vinorelbine plus cyclophosphamide plus capecitabine (VEX)) has achieved superior disease control rates (DCR) (68.6 vs. 55.6%) and progression-free survival (PFS) (median PFS of 11.1 vs. 6.9 months) as compared to MTD. The toxicity of the VEX regimen is controllable, while the toxicity of paclitaxel is more severe. The VEX group did not experience the serious adverse reaction [13]. Mechanistic studies revealed that the remarkable therapeutic outcomes of MCT could attribute to the notable effects on inhibiting angiogenesis and bolstering anti-tumor immune response [12, 14–16]. Therefore, the combination of MCT with ICI in neoadjuvant therapy may exert synergistic effects, potentially enhancing therapeutic efficacy and improving safety profiles in ESCC.

Camrelizumab, a PD-1 inhibitor, has shown promising efficacy and safety in advanced ESCC, even in chemotherapy-refractory cases, as demonstrated by the ESCORT and ESCORT-1st studies [17, 18]. In this pilot study, we innovatively investigated the utility of neoadjuvant therapy combining camrelizumab with MCT for patients with advanced ESCC and conducted a comparative analysis to assess the impact of this combined regimen versus MCT alone. Our comprehensive evaluation encompassed imaging analyses, pathological assessments, postoperative surveillance, and multi-omics analyses to interpret the impact of the neoadjuvant therapy on tumor regression and TME, as well as to identify the key molecular features and predictive factors to characterize patients suitable for combined regimen or MCT only. By doing so, we aim to enhance the precision and efficacy of this therapeutic strategy, which will further contribute to the evolving landscape of ESCC treatment.

## Methods

### Patient and sample collection

We carried out a single-center two-armed clinical trial aiming in investigating the potential benefits of combined application of camrelizumab in neoadjuvant MCT as compared to MCT alone in patients with resectable ESCC, at Huashan Hospital, Fudan University, between Jan 25, 2021, and Jan 15, 2022.

Eligible patients had to meet the following criteria: (1) aged 18 years or older; (2) histologically confirmed stage cTx-3N0-2M0 (AJCC, eighth edition) resectable ESCC, an Eastern Cooperative Oncology Group performance status of 0; (3) at least one measurable or evaluable lesion according to PET Response Evaluation Criteria In Solid Tumors (PERCIST) or Response Evaluation Criteria in Solid Tumors (RECIST); (4) adequate hematology, coagulation, liver, lung, and renal function. Exclusion criteria encompassed the following: (1) nonsquamous cell cancer; (2) inoperable or metastatic ESCCs previously treated with anti-PD-(L)1 therapies; (3) presence of another previous or current malignancy; (4) potential immunotherapy intolerance ((i.) active, known, or suspected autoimmune disease requiring systemic treatment in the past 2 years; (ii.) current use of systemic immunosuppressive medication (e.g., corticosteroids > 10 mg prednisone equivalent per day) within 7 days prior to randomization, with exceptions for topical, inhaled, or physiologic replacement therapy; (iii.) uncontrolled intercurrent illness, including but not limited to symptomatic congestive heart failure (NYHA class III/IV), unstable angina pectoris, or serious uncontrolled cardiac arrhythmia; (iv.) severe interstitial pneumonia or pulmonary infection); (5) presence of active brain or leptomeningeal metastasis or autoimmune disease. Tumor samples were microscopically reviewed by two experienced pathologists at pre-treatment and during surgery. They were collected within 30 min after esophagoscopy and surgery, then snap-frozen in liquid nitrogen for subsequent multi-omics analysis.

### Study design and interventions

**Fig. 1:**
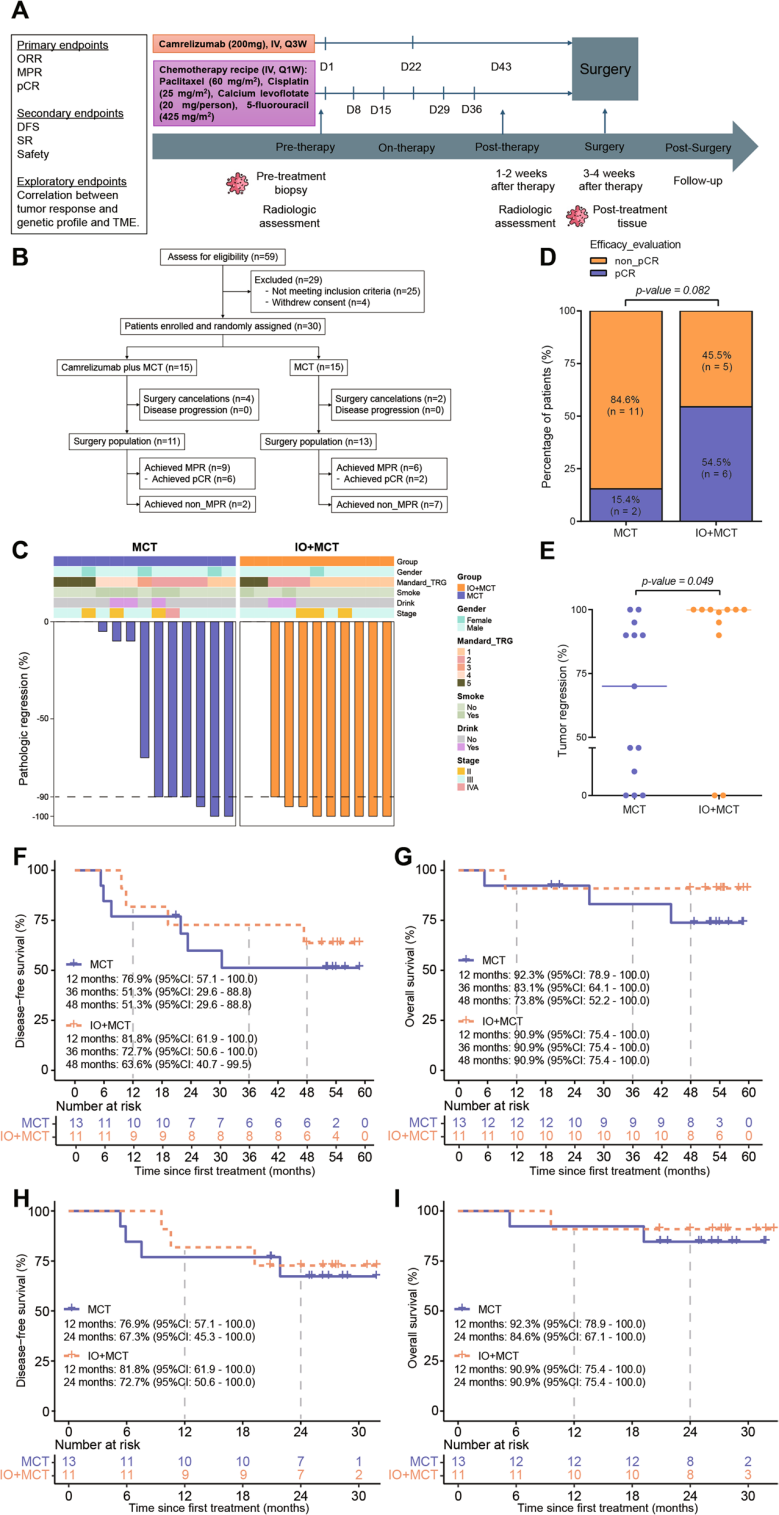
Study design and preliminary efficacy. **A** Trial schema. Eligible patients were randomly assigned to receive neoadjuvant MCT or camrelizumab plus MCT (IO + MCT), followed by surgical resection. Radiological assessments were performed before and after treatment. Tumor samples were collected at baseline and at the time of surgery. D, day of therapy. **B** Study profile. pCR, no viable tumor; MPR, 0 to 10% viable tumor. **C** Pathological response after MCT or IO + MCT treatment. Clinical and pathological features are annotated for each patient. The horizontal dashed line indicates the threshold for MPR patients. **D** The number and pathological regression rates of patients in MCT and IO + MCT. **E** Comparison between percentages of tumor regression in MCT and IO + MCT groups.** F**–**I** Kaplan–Meier curves the 4-year disease-free survival rate (**F**) and overall survival rate (**G**) for patients in MCT and IO + MCT groups. Kaplan–Meier curves the 2-year disease-free survival rate (**H**) and overall survival rate (**I**) for patients in MCT and IO + MCT groups. For **D** and **E**, *n* = 13 for MCT group, and *n* = 11 for IO + MCT group. *p* value was calculated with Fisher’s exact test and non-parametric two-sided Wilcoxon rank-sum test for the comparison of the pathological regression rates and tumor regression in MCT and IO + MCT groups

Patients meeting the eligibility criteria were randomly assigned into two groups, which received neoadjuvant chemotherapy without (termed as MCT group) or with immunotherapy (termed as IO + MCT group) before evaluation for surgical resection, respectively. Eligible patients were randomly assigned in a 1:1 ratio to receive either neoadjuvant IO + MCT or MCT alone. Randomization was performed using a computer-generated random sequence. Due to the exploratory and pilot-scale design of this study, randomization was not stratified for specific clinicopathological factors. All the patients received intravenous metronomic chemotherapy on the first day of every week for six consecutive weeks (D1, D8, D15, D22, D29, D36). The MCT regimen includes paclitaxel (60 mg/m^2^), cisplatin (25 mg/m^2^), calcium levoflotate (20 mg/person), and 5-fluorouracil (425 mg/m^2^). For the patients in IO + MCT group, the additional intravenous administrations of camrelizumab at the dose of 200 mg on D1 and D22 were performed (Fig. 1A).

Upon the completion of neoadjuvant therapy, the patients underwent clinical restaging, including PET examination, chest contrast-enhanced CT, or abdominal ultrasound, as well as physical examination, standard laboratory tests, pulmonary function tests, and nutritional assessment. Surgery was scheduled for 3 to 4 weeks after the completion of neoadjuvant therapy. Resection of the primary tumor and lymph nodes were performed in line with standard procedures for minimally invasive esophagectomy. Radiologic responses were evaluated 1 or 2 weeks after second treatment cycle, and every 3 or 6 months after surgery by two independent central expert radiologists based on PERCIST 1.0 or RECIST v1.1.

### Endpoints and response assessment and toxicity

The primary endpoint of the study was the comparison of pCR proportion, MPR proportion, and the ORR between the MCT group and the IO + MCT group. Major pathological response was defined as Mandard tumor regression grading (TRG) 2 in the primary tumor. The major pathological response also encompassed pCR, which was defined as the absence of viable tumor cells in the resected ESCC cancer specimen and all sampled regional lymph nodes. Fisher’s exact test and non-parametric two-sided Wilcoxon rank-sum test were used to calculate *p*-value and binomial distribution was used to calculate its 95% CI. Secondary outcomes included the DFS rate and OS. Additionally, disease-free survival was defined as the duration from randomization to the date of tumor recurrence or progression. Furthermore, adverse events were evaluated in all patients who had received at least two doses of the treatment and were graded according to the National Cancer Institute Common Terminology Criteria for Adverse Events, v.5.0.

### Sample size calculation

The sample size was calculated based on the primary endpoint of pCR rate. Prior to the design of this study, available literature indicated that pCR rates for neoadjuvant immunotherapy combined with chemotherapy in locally advanced ESCC ranged broadly from approximately 20 to 50% in early-phase trials [19–24]. In contrast, reported pCR rates for neoadjuvant chemotherapy alone in comparable ESCC populations were typically lower, often between 2.5 and 20% [6, 8, 23–40]. Given the exploratory and hypothesis-generating nature of this pilot study and the intention to evaluate the potential additive effect of camrelizumab, we assumed a pCR rate of 50% for the IO + MCT group and a conservative 10% for the MCT-alone group. These assumptions were designed to provide a clinically meaningful effect size to detect, while remaining within the plausible ranges reported in the literature at the time of study design. Since existing research indicated the superiority of treatment group relative to control group with respect to primary endpoints, alpha is set to be 0.05 one-sided and beta is set to be 0.2; using two-sample proportion test, the sample size is estimated to be 15 in each group.

Further methodological details, including full laboratory protocols and extended statistical analysis, are available in Additional file 1: Additional Methods [24, 32–50].

## Results

### Neoadjuvant camrelizumab plus MCT significantly improved clinical efficacy with reduced adverse effects

The study was approved by the Ethics Committee of Huashan Hospital, Fudan University (2020–837) and conducted in accordance with the Declaration of Helsinki. The trial was registered at the Chinese Clinical Trial Registry (identifier: ChiCTR2000039638). Fifty-nine patients have been enrolled and screened from January 25, 2021, to January 15, 2022. Of these patients, 4 patients withdrew consent, while 25 did not meet the inclusion criteria. Thirty patients were subsequently enrolled, then randomly assigned to receive neoadjuvant therapy with either camrelizumab (Hengrui Pharmaceuticals) combined with MCT (paclitaxel (60 mg/m^2^), cisplatin (25 mg/m^2^), calcium levoflotate (20 mg/person), and 5-fluorouracil (425 mg/m^2^)) (IO + MCT group) or MCT alone (MCT group), with 15 patients enrolled in each group. Consequently, among the 30 patients enrolled, 24 patients completed neoadjuvant therapy and underwent surgery, with 11 patients in the IO + MCT group and 13 patients in the MCT group, respectively (Fig. 1A). In the IO + MCT group, four patients did not undergo surgery including one with diagnosis of a second primary tumor (laryngeal carcinoma), one with development of liver function injury, and two cases of pulmonary infection. In the MCT-alone group, two patients did not undergo surgery including one with diagnosis of a left vocal cord soft tissue tumor and the other deciding with refusal of surgery. The demographic, clinical characteristics, and pathological variables at baseline among the enrolled patients were comparable between the two groups (Table 1). Additional file 1 Table S1 shows that there are no statistically significant differences between the two treatment groups regarding these characteristics (*p* > 0.05). However, an imbalance in baseline demographic and clinical characteristics was observed. Therefore, logistic regression analysis was conducted to assess whether factors such as age, gender, cigarette-smoking history, alcohol-drinking history, TNM stage, and tumor location had an impact on the treatment outcomes between the two groups. The forest plot for composite score regression analysis revealed that these factors did not significantly influence the treatment outcomes (Additional file 1: Fig. S1A).

**Table 1:** Baseline demographic and clinical characteristics of the included patients

	**Camrelizumab plus MCT** **(*n* = 11)**	**MCT** **(*n* = 13)**
**Age, years**	65 (58–67)	63 (54–71)
**Sex**		
Female	1 (9.1%)	3 (23.1%)
Male	10 (90.9%)	10 (76.9%)
**ECOG performance status**^A^		
0	11 (100%)	13 (100%)
1 and 2	0	0
**Cigarette-smoking history**		
Yes	2 (18.2%)	5 (38.5%)
No	9 (81.8%)	8 (61.5%)
**Alcohol-drinking history**		
Yes	2 (18.2%)	3 (23.1%)
No	9 (81.8%)	10 (76.9%)
**Tumor size stage**		
T2	1 (9.1%)	1(7.7%)
T3	10 (90.9%)	12(92.3%)
**Nodal stage**		
N0	3 (27.3%)	2 (15.4%)
N1	5 (45.5%)	6 (46.2%)
N2	3 (27.3%)	4 (30.8%)
N3	0 (0%)	1 (7.7%)
**Tumor location**		
Upper	3 (27.3%)	0 (0%)
Middle	4 (36.4%)	8 (61.5%)
Lower	4 (36.4%)	5 (38.5%)

Data are *n* (%) or median (IQR)

^A^*ECOG*, Eastern Cooperative Oncology Group

During neoadjuvant treatment, TRAEs were thoroughly evaluated and reported in all 15 patients in the IO + MCT group and in 14 out of 15 patients (93.3%) in the MCT group (Table 2). Both IO + MCT and MCT treatment demonstrated satisfactory safety profiles without grade 4–5 TRAEs, especially MCT group reported no grade 3 TRAEs. The most common grade 1 or 2 TRAEs in the IO + MCT group were lymphopenia (53.3%), electrolyte imbalance (46.7%), and hyperglycemia (46.7%), whereas that of the MCT group were electrolyte imbalance (53.3%), leukopenia (40.0%), and hyperglycemia (33.3%). Notably, three patients in the IO + MCT group (20%) experienced grade 3 TRAEs, especially lymphopenia (13.3%), in which two cases (13.3%) led to cessation of treatment. No TRAEs resulted in death. Additionally, neither neoadjuvant camrelizumab plus MCT nor MCT monotherapy was associated with any previously unreported toxic effects or severe adverse events (grade 3 or above).

**Table 2:** Summary of treatment-related adverse events in all treated patients

	**Camrelizumab plus MCT** **(*n* = 15)**^**A**^			**MCT** **(*n* = 15)**^**A**^		
	**Any grade**	**Grade 1–2**	**Grade 3**	**Any grade**	**Grade 1–2**	**Grade 3**
Any TRAES	15 (100%)	12 (80%)	3 (20%)	14 (93.3%)	14 (93.3%)	0
Adverse events leading to treatment discontinuation	2(13.3%)	0	2 (13.3%)	0	0	0
Treatment-related deaths	0	0	0	0	0	0
Leukopenia	4 (26.7%)	4 (26.7%)	0	6 (40.0%)	6 (40.0%)	0
Anemia	5 (33.3%)	5 (33.3%)	0	4 (26.7%)	4 (26.7%)	0
Thrombocytopenia	1 (6.7%)	1 (6.7%)	0	1 (6.7%)	1 (6.7%)	0
Neutropenia	7 (46.7%)	6 (40.0%)	1 (6.7%)	1 (6.7%)	1 (6.7%)	0
Lymphopenia	10 (66.7%)	8 (53.3%)	2 (13.3%)	4 (26.7%)	4 (26.7%)	0
Nausea	2 (13.3%)	2 (13.3%)	0	1 (6.7%)	1 (6.7%)	0
Blood bilirubin increased	2 (13.3%)	1 (6.7%)	1 (6.7%)	0	0	0
Elevated transaminase activity	2 (13.3%)	2 (13.3%)	0	0	0	0
Infection	1 (6.7%)	0	1 (6.7%)	0	0	0
Thyroid dysfunction	1 (6.7%)	1 (6.7%)	0	0	0	0
Electrolyte disturbance	7 (46.7%)	7 (46.7%)	0	8 (53.3%)	8 (53.3%)	0
Hyperglycemia	7 (46.7%)	7 (46.7%)	0	5 (33.3%)	5 (33.3%)	0
Elevated creatinine	2 (13.3%)	2 (13.3%)	0	2 (13.3%)	2 (13.3%)	0
Hemoptysis	1 (6.7%)	0	1 (6.7%)	0	0	0
Alopecia	1 (6.7%)	1 (6.7%)	0	0	0	0
Chronic kidney disease	2 (13.3%)	1 (6.7%)	1 (6.7%)	2 (13.3%)	2 (13.3%)	0

Data are *n* (%)

*TRAES*, treatment-related adverse events

^A^Some patients had more than one adverse event

Tumor response assessments were conducted after neoadjuvant treatment and before surgery according to the PERCIST 1.1 criteria. In the MCT group, the DCR was 100% (95% CI 77.2%, 100%) and the ORR was 84.6% (95% CI 57.8%, 95.7%), and among them, 11 cases (84.6%) and 2 cases (15.4%) achieved partial metabolic response (PMR) and stable metabolic disease (SMD), respectively (Additional file 1: Table S2). Meanwhile, in the IO + MCT group, the DCR was 100% (95% CI 74.1%, 100%) and the ORR was 90.9% (95% CI 62.3%, 98.4%). Among which, 2 cases notably (18.2%) achieved complete metabolic response (CMR), while 8 cases (72.7%) and 1 case (9.1%) demonstrated PMR and SMD, respectively. Furthermore, all patients (24/30, 80%) successfully underwent surgery, all of whom had complete tumor resection (R0). The surgically resected specimens were collected for the evaluation of pathological response (Fig. 1B). Pathological assessments revealed that a significant majority, 9 out of 11 patients (81.8%, 95%CI: 52.3%, 94.9%) in the IO + MCT group, exhibited major pathological response (MPR), with 6 patients (54.5%, 95% CI 28.0%, 78.7%) achieving pCR. Comparatively, in the MCT group, 7 out of 13 patients (53.8%, 95% CI 29.1%, 76.8%) reached MPR, and only 2 patients (15.4%, 95% CI 4.3%, 42.2%) achieved pCR (Fig. 1C, Table 3). There was a notable disparity in pCR rates between the two groups, with a crude odds ratio (OR) of 6.6 and a 95% confidential interval (CI) ranging from 0.887 to 37.59, though this did not reach statistical significance (*p* = 0.082, as shown in Fig. 1D). The median pathological regression was markedly more pronounced in the IO + MCT group at − 100%, in contrast to − 70% in the MCT group (range − 100 to − 0) (*p* = 0.049, Fig. 1E). Moreover, pathological downstaging from the pre-treatment clinical stage was observed in 18 (75%) patients, with a higher proportion in the IO + MCT group, affecting 10 out of 11 patients (90.9%) compared to 9 out of 13 patients (69.2%) in MCT group (Table 3). Using two distinct TRG scoring systems for assessment, the one-tailed Wilcoxon rank-sum test revealed a statistically significant difference between the two treatment groups according to the Mandard system (*p* = 0.042), and an even more pronounced difference when using the CAP criteria (*p* = 0.018). Table S2 provides a more comprehensive overview of the pathological outcomes for both groups. Considering the relatively small sample sizes in both treatment groups and composite primary endpoint (including pCR, MPR, and ORR), the Win Ratio method was employed to compare the primary endpoints between the two groups. The results showed that the Win Ratio for pCR was 2.29 with a 95% CI of 1.09, 6.73. Similarly, the Win Ratio for MPR was 1.78 with a 95% CI of 0.89, 4.20, and the Win Ratio for ORR was 1.13 with a 95% CI of 0.67, 1.89. Furthermore, the Win Ratio for the composite endpoints of pCR, MPR, and ORR was 2.530864 with a 95% CI of 1.01, 9.59 (Additional file 1: Fig. S1B). Additionally, the treatment effects of these three outcomes were visualized using hierarchical composite endpoints (Additional file 1: Fig. S1C), which consistently indicated that the IO + MCT group outperformed the MCT-only group.

**Table 3:** Pathologic outcome

	**Camrelizumab plus MCT** **(*n* = 11)**	**MCT** **(*n* = 13)**
**Radiographic response, *n* (%)**		
Complete response	2 (18.2)	0
Partial response	8 (72.7)	11 (84.6)
Stable disease	1 (9.1)	2 (15.8)
Progressive disease	0	0
Objective response	10 (90.9)	11 (84.6)
Disease control	11 (100)	13 (100)
**R0 resection (*n*)**	11 (100%)	13 (100%)
**Histological response of primary tumor regression grade TRG**		
^**A**^**Mandard**		
1	6 (54.5%)	2 (15.8%)
2	3 (27.3%)	5 (38.5%)
3	0	0
4	0	3 (23.1%)
5	2 (18.2)	3 (23.1%)
^**B**^**CAP**		
0	6	2
1	3	3
2	0	2
3	2	6
**ypT stage**		
ypT0	6 (54.5%)	3 (23.1%)
ypT1	3 (27.3%)	6 (46.1%)
ypT2	1 (9.1%)	2 (15.4%)
ypT3	1 (9.1%)	2 (15.4%)
ypT4	0	0
**ypN stage**		
ypN0	10 (90.9%)	6 (46.1%)
ypN1	1 (9.1%)	3 (23.1%)
ypN2	0	3 (23.1%)
ypN3	0	1 (7.7)
**LVI + PNI**^**C**^		
Positive	2 (18.2%)	6 (46.2%)
Negative	9 (81.8%)	7 (53.8%)
**ypStage**		
Including ypT0N0M0	6 (54.5%)	2 (15.8%)
ypStage I	3 (27.3%)	4 (30.8%)
ypStage II	0	2 (15.4%)
ypStage III	1 (9.1%)	4 (30.7%)
ypStage IV	0	1 (7.7%)
**Lymph node harvested, median**	0	2
**pCR**^**D**^	6 (54.5)	2 (15.4)
**MPR**^**E**^	9 (81.8)	7 (53.8)

Data are *n* (%) or median

^A^Mandard classification system

^B^*CAP*, grading system (College of American Pathologists)

^C^*LVI*, lymphovascular invasion; *PNI*, perineural invasion

^D^*pCR*, complete pathological response

^E^*MPR*, major pathological response

Postoperative complications were reported in 2 of these 24 patients; one in the IO + MCT group suffered from electrolyte imbalance, while the other in the MCT group experienced cardiac failure (Additional file 1: Table S3).

By the final data cutoff date of December 20, 2025, the median duration of follow-up for surviving patients was 53.3 months (range from 5.3 to 59.7 months). In the updated survival analysis, the 4-year DFS and OS rates for the IO + MCT group were 63.6% (95% CI, 40.7 to 99.5) and 90.9% (95% CI, 75.4 to 100), respectively. For the MCT group, the corresponding 4-year rates were 51.3% (95% CI, 29.6 to 88.8) for DFS and 73.8% (95% CI, 52.2 to 100) for OS (Fig. 1H, I). Notably, one patient in the IO + MCT group died of a non‑cancer cause (aspiration on postoperative day 2), which contributed to an OS event unrelated to oncologic treatment efficacy. For reference, the earlier interim analysis (data cutoff October 1, 2023; median follow-up 26.3 months) reported 2-year DFS and OS rates of 72.7% and 90.9% in the IO + MCT group, and 67.3% and 84.6% in the MCT group, respectively (Fig. 1F, G). Patients who achieved a pCR continued to show a trend toward longer DFS and OS compared to non‑pCR patients (Additional file 1: Fig. S2A‑S2D). All randomly assigned patients were included in the intention‑to‑treat (ITT) analysis for OS regardless of treatment completion (Additional file 1: Fig. S2E, S2F). In the updated ITT analysis, the 4‑year OS rates were 66.7% (95% CI, 46.6–95.3) for the IO + MCT group and 70.0% (95% CI, 49.2–99.7) for the MCT group, with no statistically significant difference between groups.

### Neoadjuvant camrelizumab combined with MCT reprogrammed the immune-suppressive tumor microenvironment

To elucidate the underlying mechanisms regarding the superior efficacy observed in the IO + MCT group compared to the MCT group, we initiated our investigation with bulk RNA sequencing (RNA-seq) analyses. This involved comparing paired samples of pre-treatment tumor biopsies and post-treatment surgical resections from 17 patients, with 11 from the MCT group and 6 from the IO + MCT group. A total of 2490 differentially expressed genes (DEGs) (1292 upregulated and 1198 downregulated) in the IO + MCT group and 1196 DEGs (438 upregulated and 758 downregulated) in the MCT group were identified when comparing the paired post-treatment to pre-treatment samples in the separate groups (Additional file 1: Fig. S3A). The Venn diagram uncovered distinct transcriptome alterations induced by the different therapeutic approaches (Additional file 1: Fig. S3B). Pathway enrichment analysis of the DEGs showed that muscle contraction and cell cycle checkpoints were the most significantly enriched pathways for up- and downregulated DEGs in post-treatment versus pre-treatment samples in the IO + MCT group (Additional file 1: Fig. S3C, D). Meanwhile, extracellular matrix organization and neutrophil degranulation were the predominantly enriched pathways for the MCT group (Additional file 1: Fig. S3E, F). Moreover, gene set variation analysis (GSVA) of hallmark gene sets indicated that several pathways including those involved in apical surface in cellular components, angiogenesis, myogenesis, NOTCH, and Hedgehog signaling exhibited significantly enhanced expression in post-treatment samples of both the IO + MCT and MCT groups. These commonalities are likely reflective of the effects of MCT (Additional file 1: Fig. S3G). Notably, discernible differences between the two neoadjuvant treatment regimens were observed in the paired resection specimens. A direct comparison of post- versus pre-treatment samples in the IO + MCT group highlighted specific enrichment of gene sets such as apical junction in cellular components, bile acid metabolism, androgen and estrogen response, IL-2/STAT5 signaling, and Wnt/β-catenin signaling. In contrast, the MCT-only group showed an upregulation of epithelial-mesenchymal transition (EMT) in post-treatment samples, which was reversed by immunotherapy.

**Fig. 2:**
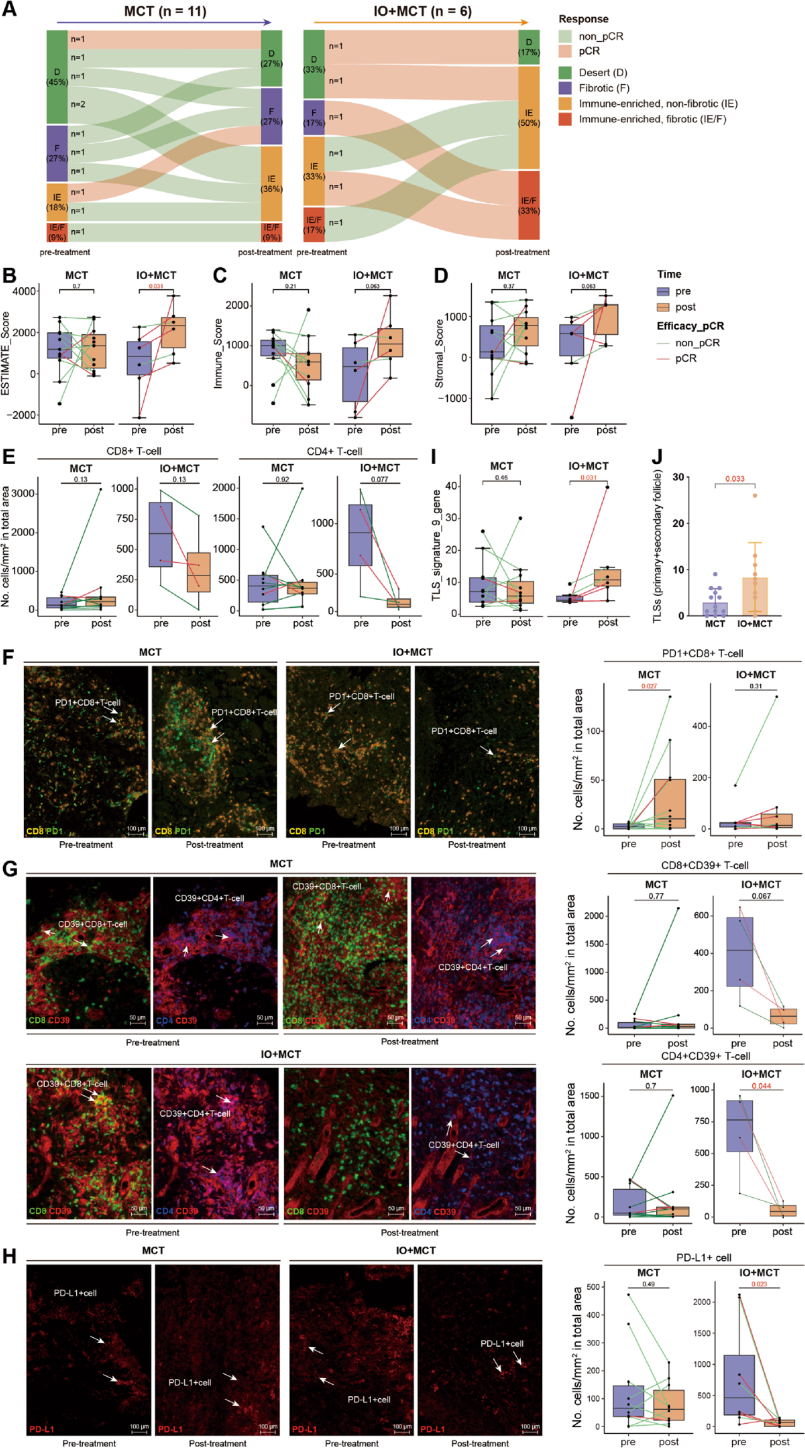
Neoadjuvant camrelizumab plus MCT reprogrammed cold tumor microenvironment. **A**–**D** Paired samples of pre-treatment tumor biopsies and post-treatment surgical resections from 17 patients, with 11 from the MCT group and 6 from the IO + MCT group collected for RNA-seq and subsequent analyses.** A** Dynamic of TME subtypes in ESCC patients during treatment with MCT only or camrelizumab plus MCT (IO + MCT). **B**–**D** Changes of estimate score (**B**), immune score (**C**), and stroma score (**D**) in both MCT and IO + MCT groups before and after treatment.** E**–**H** mIF staining was performed to assess the changes of the density of CD4 + T cells and CD8 + T cells (**E**), PD1 + CD8 + T cells (**F**), CD4 + CD39 + (**G**), PD-L1 + cells (**H**) in both MCT and IO + MCT groups before and after treatment.** I** TLS signature score comparison between MCT and IO + MCT group were calculated based on the RNA-seq results. **J** The number of primary and secondary follicle-like TLSs were counted for analyses based on the staining of pathological tissue sections of surgical resections. **K** Representative images of mIF staining on tissue sections from pCR (Mandard TRG1) and non-pCR (Mandard TRG5) patients after IO + MCT treatment. Syto13 (blue), PanCK (green), CD3 (purple), and CD20 (yellow). *p* value was calculated using a two-sided paired Wilcoxon rank-sum test for comparison of the TME-related signature and cell scores in the pre- and post-treatment specimen, while a non-parametric two-sided Wilcoxon rank-sum test was performed for the comparison of TLS score in MCT and IO + MCT groups

As per previous research highlighting the immune-response modulating capabilities of MCT [16, 51, 52], we conducted an assessment of the alterations in tumor microenvironment (TME) phenotype and the composition of tumor-infiltrating immune cells through RNA-seq. Here, following neoadjuvant IO + MCT treatment, among the non-immune-enriched (IE) subtype which include fibrotic (F) and immune desert (D) phenotypes, 66.7% (2 out of 3) demonstrated a noticeable inclination toward an anti-tumor response akin to the IE subtype, characterized by immune-enriched non-fibrotic (IE), and immune-enriched fibrotic (IE/F) features. In contrast, in the MCT group, only 37.5% (3 out of 8) of non-IE tumors underwent a transition to IE tumors, without achieving a complete pathological response (Fig. 2A). In addition, we analyzed the changes in tumor-infiltrating immune cells, noting a consistent upward trend in the estimate score (*p* = 0.031), immune score (*p* = 0.063), and stroma score (*p* = 0.063) in the IO + MCT group but not in MCT group (Fig. 2B, C, D). These results imply that neoadjuvant immunotherapy combined with MCT can alter the immune profile of tumors, transforming them from immune-suppressive to immune-active environments.

To further substantiate these findings, multiplex immunofluorescence (mIF) staining was performed to detect PD-L1 along with other immune biomarkers, including helper T cell marker CD4, cytotoxicity T cell marker CD8, and exhausted markers PD-1 and terminal exhausted markers CD39, in paired pre-treatment tumor biopsies and post-treatment surgical resections. PanCK was used to delineate the tumor region, with marker densities in the PanCK-positive tumor and stromal regions being assessed independently. Immune cell counts were conducted in both the stromal and tumor regions in non-pCR patients. For cases achieving pCR after therapy, only stromal regions were scored due to the absence of residual viable tumor cells. No significant changes were observed in the overall density of CD8 + or CD4 + T cells as a result of the treatment in either group (Fig. 2E). However, there was a significant increase in the density of PD-1 + CD8 + T cells in the MCT group, a phenomenon not observed in the IO + MCT group (Fig. 2F), implying that anti-PD-1 therapy may counteract the accumulation of PD-1 + CD8 + T cells induced by MCT therapy. Additionally, co-localization of CD39 with CD4 or CD8 was observed in both groups. Further analyses revealed a significant decrease in the densities of CD39 + CD8 + and CD39 + CD4 + T cells in response to IO + MCT treatment compared with MCT group (Fig. 2G), further underscoring the depletion of exhausted T cells in the IO + MCT group. Notably, a marked reduction in the infiltration of PD-L1 + cells was observed post-treatment in the IO + MCT group (Fig. 2H). Concurrent analyses of RNA-seq data from both pre- and post-treatment groups revealed shifts in the pattern of immune cells (Additional file 1: Fig. S4A). A significant enhancement of the abundance of effector memory CD4 + T cells, effector memory CD8 + T cells, immature dendritic cells, and activated B cells was observed in the IO + MCT group (Additional file 1: Fig. S4B–E), suggesting the substantial activation of immune cells. The remarkable activation of B cells prompted us to further look into the tertiary lymphoid structures’ (TLSs) signature score, which exhibited a significant increase in the IO + MCT group post-neoadjuvant treatment, indicating maybe the formation of more TLSs at the primary tumor cites of pCR patients (*p* = 0.031, Fig. 2I). The pathological staining of post-treatment surgical resections of the two groups revealed a higher number of primary and secondary follicle-like TLSs in the IO + MCT group (Fig. 2J).

### The downregulation of mitochondrial respiratory chain-related genes and pathways is a key feature for well-responders in IO + MCT group

**Fig. 3:**
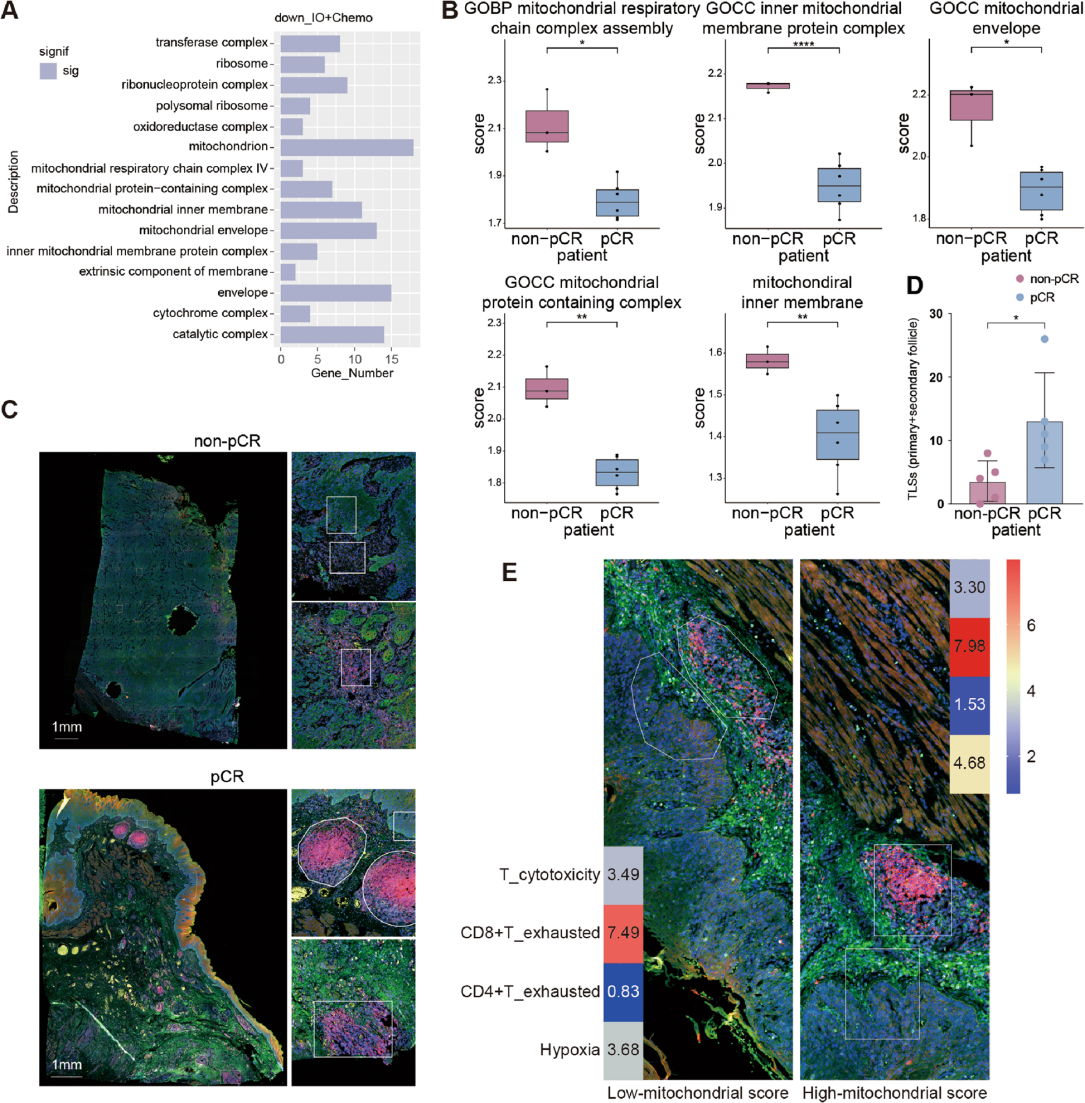
The downregulated mitochondrial function is a key feature for well-responders in IO + MCT group. **A** Functional enrichment analysis of the downregulated genes between pre-treatment samples of patients with pCR and non-pCR in the IO + MCT group based on the RNA-seq results. **B**–**D** Representative tissue sections from three patients (one pCR patient of Mandard TRG1 and two non-pCR patients of Mandard TRG2 and TRG5) in the IO + MCT group were subjected to DSP-WTA with multiple ROIs selected. **B** ssGSEA scores of the mitochondrial gene sets from the GSEA database were calculated based on the DSP-WTA results of the tumor compartment ROIs to display the differences between ROIs in the pre-treatment samples of non-pCR and pCR patients. **C** Representative mIF staining images of tissue sections of non-pCR and pCR patients. **D **The comparison of the number of primary and secondary follicular-like TLSs in the tissue sections from non-pCR and pCR patients. **E** The two TLS ROIs adjacent to the tumor ROIs with distinct mitochondrial scores were selected for evaluation at the post-treatment section from the patient with Mandard TRG2. The scores of T-cell cytotoxicity, CD8 +/CD4 + T cell exhaustion, and hypoxia were calculated for comparison. Syto13 (blue), PanCK (green), CD3 (purple), and CD20 (yellow). *p* value was calculated with non-parametric two-sided Wilcoxon rank-sum test and paired two-sided Wilcoxon rank-sum test for the comparison of the mitochondrial gene sets scores of the representative tissue sections from three patients

To enhance the predictive capacity for patient response to IO + MCT, we further performed the comparative transcriptomic analysis of pre-treatment samples of two groups and correlated the findings with the clinical outcomes. This analysis identified 571 and 1267 DEGs between pre-treatment samples of patients with pCR and non-pCR in the IO + MCT and MCT groups, respectively (Additional file 1: Fig. S5A, B). Functional enrichment analysis of the upregulated genes in the IO + MCT and MCT groups demonstrated that the upregulated genes in the IO + MCT group were primarily enriched in lysosome, Golgi apparatus, and apical cell parts, while those in the MCT group were mainly involved in the biological processes of nuclear lumen, nucleoplasm, and vesicle (Additional file 1: Fig. S5C). Intriguingly, patients in the IO + MCT group who achieved pCR exhibited significantly reduced expression of genes linked to the mitochondrial and envelope functions (Fig. 3A).

To investigate the potential role of mitochondrial function in the efficacy of IO + MCT group, we conducted digital spatial profiling-whole transcriptomic analysis (DSP-WTA) on the representative pre- and post-treatment tissue sections from 3 patients with varying clinical outcomes, including pCR (one patient of Mandard TRG1) and non-pCR (two patients of Mandard TRG2 and Mandard TRG5). The tissue sections were pre-stained with Syto13 (blue), PanCK (green), CD3 (purple), and CD20 (yellow) to assess morphology, with regions of interest (ROIs) selected for subsequent whole transcriptomic analysis. The expression levels of genes in the mitochondrial gene set were extracted from these tumor samples for each patient. Single-sample Gene Set Enrichment Analysis (ssGSEA) was then applied to calculate the enrichment scores for each tumor region derived from the pre-treatment samples of non-pCR and pCR patients, providing a quantitative measure of mitochondrial activity. The results indicated that the ssGSEA scores of mitochondrial respiratory chain (MRC)-related pathways in pCR patients were significantly lower than those in non-pCR patients, including mitochondrial respiratory chain complex assembly, inner mitochondrial membrane protein complex, mitochondrial envelope, mitochondrial protein containing complex, and mitochondrial inner membrane (Fig. 3B). These indicated that patients with lower MRC scores may experience better outcomes with combination therapy, highlighting the importance of mitochondrial scoring in predicting the efficacy of chemotherapy combined with immunotherapy. Furthermore, mIF staining results indicated that IO + MCT treatment significantly promoted immune infiltration, particularly TLS formation and maturation in pCR patient sample (Fig. 3C). Additional validations via pathological staining confirmed that the number of primary and secondary follicular-like TLS in pCR cases was significantly higher than that of the non-pCR cases following IO + MCT treatment (*p* = 0.019, Fig. 3D). We further explored the relationship between tumor MRC score and TLS in the tumor microenvironment. In a patient with Mandard TRG2 after IO + MCT treatment, we observed that the exhaustion scores of CD4 + T cells and CD8 + T cells in the TLS region adjacent to the tumor compartment with lower MRC scores were less than those in the TLS region adjacent to the tumor compartment with higher MRC scores, and the hypoxia score was also decreased (Fig. 3E). In addition, DSP-WTA analysis revealed a significant reduction in the expression of the terminal exhaustion marker CD39 in tumor-infiltrating T cells following IO + MCT treatment in both non-pCR and pCR patients (Additional file 1: Fig. S6). These indicate that IO + MCT treatment effectively alleviates immune cell exhaustion and activates TLS, reinforcing its potential to modulate the immune microenvironment.

### IRMCS score as a potential response biomarker for IO + MCT

**Fig. 4:**
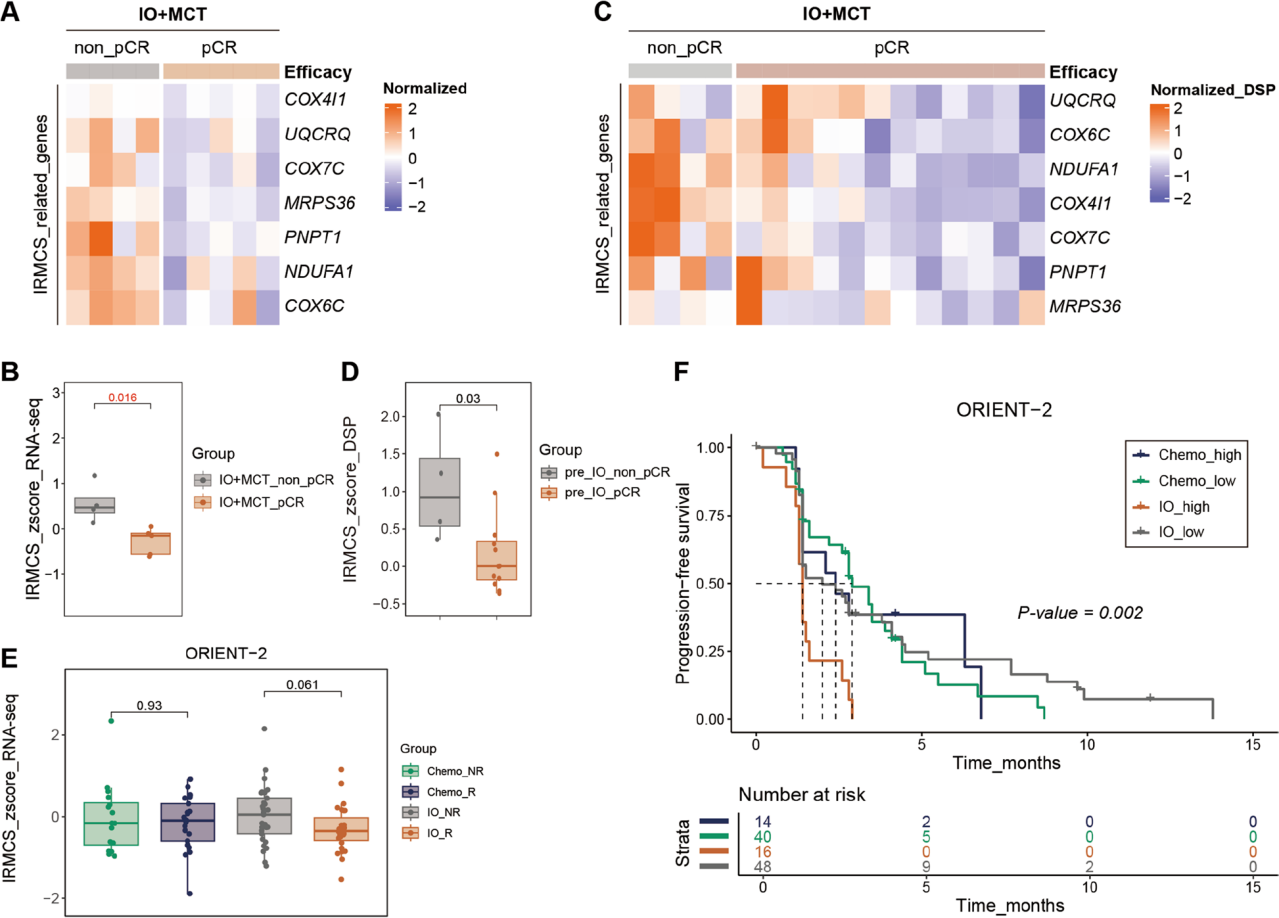
IRMCS score as a potential response biomarker for IO + MCT. **A**/**C** Heatmap of differential expression of seven genes (*MRPS36*, *COX4I1*, *COX7C*, *COX6C*, *PNPT1*, *UQCRQ*, and *NDUFA1*) associated with the mitochondrial protein-containing complex between pre-treatment samples of pCR and non-pCR patients in the IO + MCT group with the RNA-seq analysis (**A**) or DSP-WTA analysis (**C**). **B** The Immuno-oncology Related Mitochondrial Complex Signature (IRMCS) scores were calculated using the ssGSEA algorithm for comparison between pCR and non-pCR patients in the IO + MCT group with RNA-seq results. **D** IRMCS_zscores were calculated using the ssGSEA algorithm for comparison between pCR and non-pCR patients in the IO + MCT group with DSP-WTA results. **E** Verification of the predictive value of IRMCS score in the ORIENT-2 cohort between responders (R) and non-responders (NR). **F** The correlation between IRMCS scores and prognosis in the ORIENT-2 cohort was examined. *p* value was calculated using a non-parametric two-sided Wilcoxon rank-sum test for comparison of the IRMCS score between pCR (R) and non-pCR (NR) group, while a log-rank test for the progression-free survival rate

Given the significance of the mitochondrial pathway in the context of IO + MCT group as mentioned above, we conducted an in-depth analysis to identify the specific differential genes related to this pathway with the bulk RNA-seq data. Our analysis revealed a significantly reduced expression of seven genes (*MRPS36*, *COX4I1*, *COX7C*, *COX6C*, *PNPT1*, *UQCRQ*, and *NDUFA1*), which are associated with the mitochondrial respiratory chain and oxidative phosphorylation in pCR patients within the IO + MCT group (Fig. 4A) [41–47]. Based on this finding, we established the Immuno-oncology Related Mitochondrial Complex Signature (IRMCS) and calculated the signature score utilizing the ssGSEA algorithm. Strikingly, pCR patients in the IO + MCT group exhibited a significantly lower IRMCS score than those of non-pCR (Fig. 4B). This correlation suggests that a lower IRMCS score may be indicative of a more favorable response to IO + MCT therapy. To substantiate these results, we reassessed the IRMCS scores using DSP-WTA, which corroborated the findings from the bulk RNA analysis. DSP-WTA results confirmed that the IRMCS scores of pCR patients were significantly lower than those of non-pCR patients in IO + MCT group (Fig. 4C, D). Subsequently, we investigated the correlation between the IRMCS score and the prognostic outcomes of immunotherapy in advanced esophageal cancer by utilizing public databases. In the ORIENT-2 cohort, patients who exhibited a response to immunotherapy (R, PR + SD) had lower IRMCS scores than non-responders (NR, PD) (Fig. 4E). Moreover, patients with lower IRMCS scores experienced significantly shorter PFS following immunotherapy compared to those with higher scores (Fig. 4F). This association further underscores the value of the IRMCS score in predicting the efficacy of ICI. In summary, our findings propose that the IRMCS scores could serve as a predictive biomarker for the efficacy of immunotherapy combined with chemotherapy in ESCC. This biomarker has the potential to significantly inform clinical decision-making processes and guide the development of personalized treatment strategies.

### Correlation between genomic features and pathological response

Meanwhile, of the 24 patients who underwent surgery, 22 had sufficient primary tumor samples prior to treatment that were suitable for targeted DNA sequencing. The most commonly mutated gene was *TP53* (77%), followed by *NOTCH1* (50%), aligning with previous ESCC cohorts (Additional file 1: Fig. S7A). However, the analysis did not uncover any enriched mutations specifically in pCR patients in neither IO + MCT nor MCT group. Similarly, no significant differences were observed in tumor mutation burden (TMB) and mutant-allele tumor heterogeneity (MATH) score between patients with pCR and non-pCR (Additional file 1: Fig. S7B, C).

**Fig. 5:**
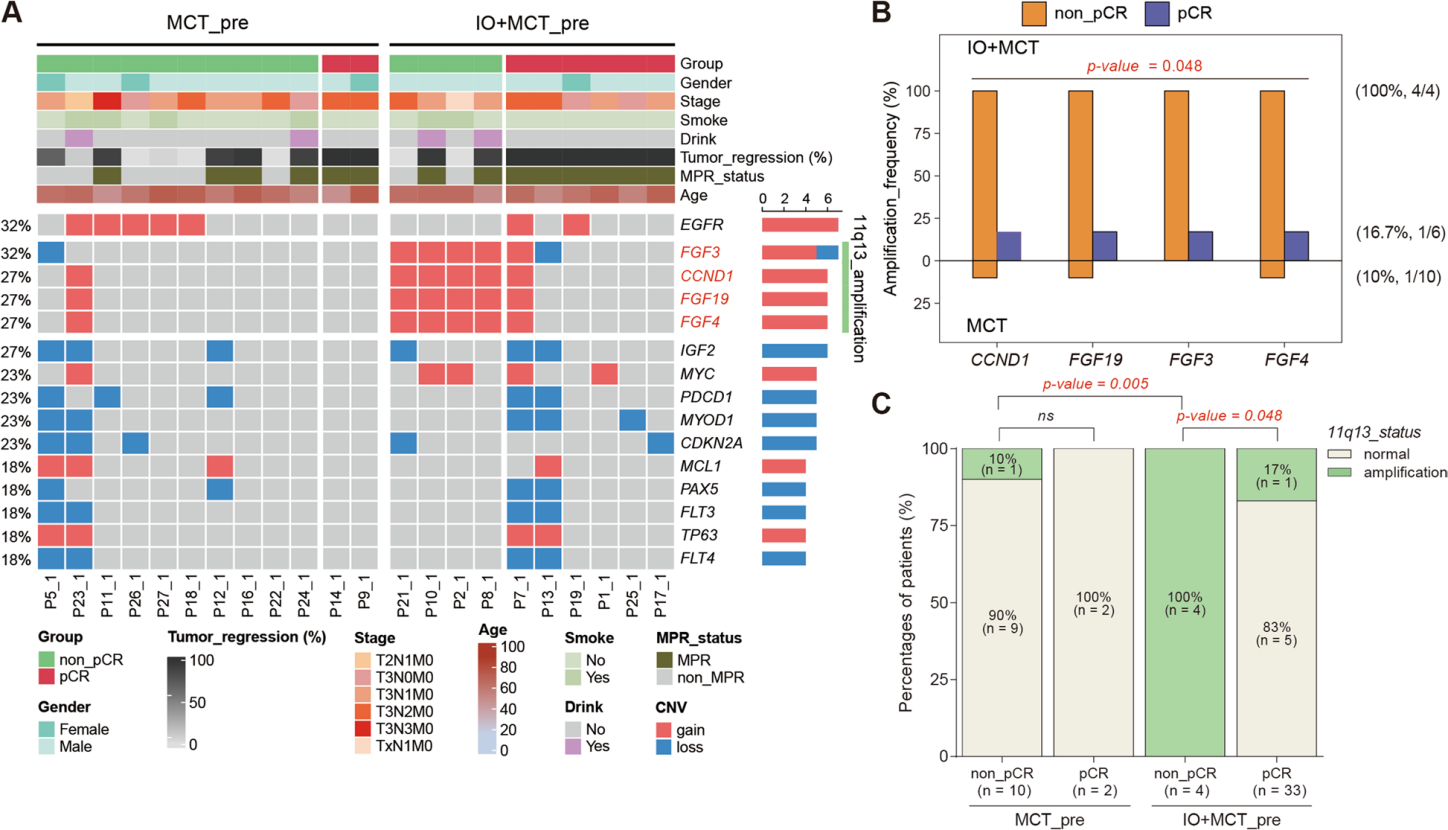
Correlation between genomic amplification and pathological response. **A** Frequency of common genetic amplifications in ESCC patients. The aberrant amplifications of *CCND1*, *FGF3*, *FGF19*, and *FGF4* were identified in the non-pCR patients of IO + MCT group. All these four genes are located in the chromosome 11q13 region. **B** The ratios of aberrant *CCND1*, *FGF3*, *FGF19*, and *FGF4* amplifications in patients with and without pCR in the IO + MCT group. **C** Comparative analysis of the frequency of 11q13 amplifications among non-pCR and pCR patients in the IO + MCT and MCT group. *p* value was calculated with Fisher’s exact test

Finally, we investigated the correlation between copy number variation and pathological response. Amplifications of *EGFR*, *CCND1*, *FGF3*, FGF19, and *FGF4* were identified as recurrent alterations in ESCC patients (Fig. 5A). In the IO + MCT group, all non-pCR patients displayed concurrent amplifications of *CCND1*, *FGF3*, *FGF19*, and *FGF4*, which were located in the chromosome 11q13 region (Fig. 5B). Notably, the prevalence of amplification in this region was markedly higher in patients without pCR (100%) than in those with pCR (17%), suggesting a potential link between 11q13 amplification and the likelihood of complete pathological regression when camrelizumab is administered in combination with MCT (Fig. 5C).

## Discussion

In this cohort of patients with locally advanced resectable ESCC, the preoperative combination of camrelizumab (anti-PD-1) with low-dose metronomic chemotherapy (IO + MCT) resulted in a clinically meaningful increase in the pCR rate compared to MCT alone. Achieving pCR is a validated surrogate endpoint associated with improved long-term survival [53, 54]. To contextualize our findings, the pCR rate achieved with IO + MCT (54.5%) compares favorably with rates reported in pivotal trials establishing current standards of care, including the CROSS trial (49% in the ESCC subgroup), the NEOCRTEC5010 trial (43.2%), and contemporary chemoimmunotherapy studies such as the NICE trial (42.5%) and KEYSTONE-001 (41.4%) [55–59], as well as those from other neoadjuvant chemoimmunotherapy studies in ESCC [6, 8, 19–23, 25–31, 59–63] (Additional file 1: Fig. S8). All 24 patients successfully completed planned surgery with a 100% R0 resection rate, consistent with outcomes from similar neoadjuvant immunotherapy-chemotherapy trials such as the NICE trial [64] and NCT04460066 trial [28], collectively demonstrating the robust antitumor activity of this combination. With a median follow-up of 53.3 months, the IO + MCT group achieved a 4-year OS rate of 90.9% and a 4-year DFS rate of 63.6%. These outcomes compare favorably with historical benchmarks (e.g., approximately 75–80% 2-year OS/DFS in the ESCC subgroup of the CROSS trial), with encouraging survival signals from modern immuno-chemotherapy regimens. In the ITT analysis which included all randomly assigned patients, the 4-year OS rates were 66.7% for the IO + MCT group and 70.0% for the MCT group, with no statistically significant difference. The lower OS in the ITT analysis, compared to the per-protocol analysis, primarily reflects the impact of events unrelated to oncologic efficacy. These include one non-cancer-related postoperative death in the IO + MCT group and treatment discontinuations due to intercurrent events (e.g., second primary tumors or infections) in both groups. The convergence of long-term OS rates in the ITT analysis may also be influenced by the efficacy of subsequent salvage therapies received by patients who experienced disease progression, which could attenuate survival differences between the initial treatment arms.

Meanwhile, the combination of camrelizumab to neoadjuvant chemotherapy not only significantly enhances pathological response but is also associated with promising long-term survival outcomes in our cohort. Nonetheless, given the limited sample size of this pilot study, the current analysis remains underpowered to detect a statistically significant survival difference between treatment groups. Definitive confirmation of survival benefit will require validation in large-scale phase III trials.

MCT, an alternative to traditional MTD chemotherapy, delivers drugs at lower doses over a prolonged period of time, typically on a continuous or frequent schedule without extended breaks. This approach aims to minimize the toxicity associated with high-dose chemotherapy while maintaining therapeutic efficacy. A key mechanism underpinning the effectiveness of MCT is its anti-angiogenic activity, which suppresses the formation of new blood vessels that are essential for tumor growth. Additionally, MCT reduces the likelihood of developing drug resistance. Unlike MTD chemotherapy which can lead to the selection of resistant cancer cell clones, the continuous low-dose pressure of MCT may help mitigate such resistance. Furthermore, MCT exhibits immunomodulatory functions that can potentially produce a synergistic effect with immunotherapy. Studies have shown that MCT promotes immune cell infiltration and decreases immunosuppressive elements within TME, thus improving therapeutic response.

Our study has revealed that the neoadjuvant IO + MCT combination synergistically reprograms the TME, notably by upregulating specific immune cell subsets including effector memory CD4/8 T cells and DC cells. While CD8 + T lymphocytes are pivotal in anti-PD-1-based immunotherapies, the mere presence of CD8 + T cell within tumors does not necessarily correlated with therapeutic efficacy of immunotherapy [65]. Concurrently, an increased number of primary and secondary follicle-like TLS have been observed, indicating a more robust immune response, which was associated with better prognosis. These mature TLS may even predict the efficacy of ICI, independent of PD-L1 expression levels [66, 67]. Thus, our data suggests that the beneficial efficacy of IO + MCT may attribute to the promotion of a favorable, TLS-enriched, memory T-cell-biased microenvironment. This hypothesis provides a strong rationale for future functional studies using preclinical in vivo models to establish potential causality. Furthermore, our findings indicate that a substantial proportion of CD8 + T cells in the TME may be bystander cells that lack tumor reactivity [68]. Notably, the IO + MCT regimen has been shown to disrupt the abundance of PD1 + CD8 + cells which are the target cells of anti-PD-1 therapy, while also significantly downregulates CD39 + CD8 + cells. CD39, which is co-expressed with several inhibitory receptors on CD8 + T cells including LAG3, TIGIT, PD1, TIM3, and 2B4, is a marker often associated with T cell exhaustion [69]. The decrease in intratumoral CD39 + CD8 + T cells suggests that IO + MCT may alleviate the exhaustion of CD8 + T cells. These CD39 + CD8 + T cells have been shown to react to neoantigens and tumor-associated antigens in lung cancer and melanoma [70, 71]. Their presence in the baseline tumor samples suggested that there are effector CD8 + cells capable of recognizing tumor neoantigen and tumor-associated antigen. The absence of these cells within tumors implies a lack of tumor-specific CD8 + T cells, potentially due to a defect in the cancer-immunity cycle. These observations provide valuable insights into the impact of neoadjuvant therapy on ESCC TME and implications for tumor-specific CD8 + T cells.

Another notable discovery in our study was the differential activity of MRC-related pathways among pCR and non-pCR patients in the IO + MCT group. pCR patients exhibited significantly lower MRC scores across various mitochondrial pathways, suggesting that a reduction in MRC activity may correlate with a more favorable response to the combination therapy of IO + MCT. MRC is a central process to oxidative phosphorylation (OXPHOS), which plays a critical role in meeting the bioenergetic and macromolecular anabolic needs of cancer cells in many tumor types [72]. In our study, the lower MRC scores observed in pCR patients may reveal the potential vulnerability of tumors, as reduced mitochondrial function could restrict tumor growth and metastatic potential. This observation is in line with prior studies emphasizing the critical role of mitochondria in biosynthesis, energy production, and tumor progression [73]. A reduction in MRC activity compromises tumor cells’ ability to acquire energy acquisition, impairing their metabolic adaptability and increasing their susceptibility to neoadjuvant therapies. Additionally, the interplay between mitochondrial activity and the immune microenvironment is particularly intriguing. An elevated MRC score in tumor sites may contribute to hypoxic TME, a condition known to induce T-cell exhaustion. Hypoxia-induced T-cell exhaustion is characterized by impaired T-cell effector functions, upregulated inhibitory receptor expression, and reduced cytokine production, all of which can diminish anti-tumor immunity. Conversely, in pCR patients with lower MRC activity, the less hypoxic TME may support more effective T-cell function and a robust immune response, thereby augmenting the efficacy of IO + MCT therapy. Several clinical studies are currently exploring the targeting mitochondrial metabolism for cancer treatment. For example, Metformin and Phenformin inhibit mitochondrial Complex I, while Atovaquone inhibits Mitochondrial Complex III. These complexes are essential components of the MRC [74–76]. These strategies aim to disrupt the energy supply to tumor cells, thereby inhibiting their growth. Therefore, targeting mitochondrial function could represent a promising strategy for improving the efficacy of combination therapies in ESCC. As a pilot investigation, these TME observations are inherently exploratory given the limited sample size and warrant future validation in expanded cohorts.

The pioneering outcome of our exploratory biomarker analysis was the identification of the Immuno-oncology Related Mitochondrial Complex Signature (IRMCS), which encompasses seven genes associated with mitochondrial function: *MRPS36*, *COX4I1*, *COX7C*, *COX6C*, *PNPT1*, *UQCRQ*, and *NDUFA1*. Notably, the IRMCS scores were found to be significantly lower in the pre-treatment samples of pCR patients as compared to non-pCR patients in the IO + MCT group. This suggests that the IRMCS score is a negative predictor of the therapeutic response, aligning with the finding that reduced mitochondrial respiratory chain and oxidative phosphorylation correlates with enhanced treatment efficacy. Furthermore, this association was corroborated in the ORIENT-2 cohort [48], where patients presenting with lower IRMCS scores were observed to have better response to immunotherapy. The IRMCS embodies an integrated biomarker paradigm, supporting the concept that effective anti-PD-1 therapy requires both a favorable immune contexture and a receptive tumor metabolic state. A key interpretive consideration is its derivation from bulk tumor RNA sequencing. While this approach captures the global transcriptional state of the tumor-immune ecosystem, it inherently conflates signals from malignant cells, diverse immune infiltrates (T cells, B cells, macrophages, etc.), and stromal components. Therefore, the IRMCS reflects a composite phenotype of the TME. The “mitochondrial complex” component likely stems predominantly from tumor cells, whereas the “immuno-oncology” component is primarily driven by immune cells. Consequently, the predictive strength of IRMCS may arise from a favorable alignment or interaction between a tumor with low oxidative metabolic activity and an infiltrating, pre-activated immune milieu, rather than from either factor alone. This integration may constitute its strength as a biomarker. However, it precludes definitive deconvolution of cellular contributions within the current data. Future validation studies employing single-cell RNA sequencing or spatially resolved transcriptomics on pre-treatment biopsies would be essential to resolve the signature’s cellular origins and refine its biological interpretation.

In our exploratory genomic analysis, amplification of the 11q13 locus (encompassing *CCND1*, *FGF3*, *FGF19*, and *FGF4*) was enriched in non-pCR patients. This finding aligns with previous studies that unresectable hepatocellular carcinoma patients with Amp11q13 are less likely to benefit from PD-1 blockade therapies [77] and to resistance to immune checkpoint inhibitors in nasopharyngeal carcinoma [23]. A recent molecular classification based on the multi-omics analysis has categorized ESCC with *CCND1* amplification into a cell cycle pathway activation subtype [78]. Therefore, 11q13 amplification represents a biologically plausible candidate resistance mechanism of the IO + MCT regimen in our cohort. However, we explicitly note that this observation is derived from limited sample size, compromising statistical confidence and precluding definitive conclusions. It should therefore be regarded as a hypothesis-generating finding that warrants validation in larger cohorts. Given that the CDK4/6 inhibitor Palbociclib, which targets cell cycle alterations, has been approved for the treatment of breast cancer [1], it is plausible that ESCC exhibiting such cell cycle alterations might be susceptible to palbociclib. Future studies should investigate this potential therapeutic vulnerability.

In contrast to the 11q13 findings, we observed no significant differences in tumor mutational burden (TMB), mutation frequency of common genes, or MATH scores (a measure of intratumor heterogeneity) between response groups. While these factors are established biomarkers in some immunotherapy contexts, their predictive value in ESCC, particularly within neoadjuvant chemoimmunotherapy, remains less defined. Importantly, the lack of a statistically significant association in our study cannot exclude their clinical and biological relevance. Given our limited cohort size, the analysis was inherently underpowered to detect modest but potentially clinically meaningful differences in these continuous or low-frequency genomic features. This highlights the challenge of drawing definitive negative conclusions from pilot-scale genomic studies.

Several limitations of our study should be duly recognized. First, the modest sample size (*N* = 24) limits the statistical power for subgroup analyses and underscores the need to validate the observed high pathological complete response (pCR) rate in larger cohorts. Although baseline characteristics were balanced, the absence of stratified randomization in this small trial means that undetected confounding cannot be ruled out. Second, the single-center, single-ethnicity design may affect the generalizability of our findings to broader, more diverse populations. Future multi-center studies with larger sample sizes are essential to confirm and extend our preliminary conclusions. Third, our study was designed to evaluate short-term efficacy, which underscores the necessity for extended follow-up studies to determine OS and DFS and further assessment of the enduring clinical benefits of neoadjuvant IO plus MCT for locally advanced ESCC. Besides, although the treatment combination demonstrated acceptable tolerability without new safety signals, the limited cohort size and follow-up duration preclude comprehensive assessment of rare, severe, or delayed-onset immune-related adverse events. Larger prospective studies with extended follow-up are required to establish the long-term safety profile of this neoadjuvant IO + MCT regimen. At last, while our multi-omics profiling revealed significant TME reprogramming associated with treatment response, these findings remain correlative. The functional properties of identified immune subsets (e.g., CD39 + CD8 + T cells) and a direct causal relationship between specific TME features (e.g., TLS formation) and pCR achievement were not experimentally validated in this study and warrant further mechanistic investigation.

Despite these limitations, the combination of camrelizumab with a low dose MCT in neoadjuvant settings for surgically resectable ESCC has demonstrated satisfactory antitumor activity. A significant proportion of patients were able to undergo R0 surgical resection and achieve pCR. The regimen exhibited a manageable safety profile within the observed follow-up period. To validate and extend these findings, larger, prospective and ideally randomized phase II/III trials with extended follow-up are warranted. Such studies should aim not only to confirm the efficacy and long-term safety but also to prospectively validate the predictive biomarkers (e.g., IRMCS) and resistance mechanisms (e.g., 11q13 amplification) identified here. The insights from this work could ultimately contribute to a more tailored, biomarker-guided treatment strategy, optimizing therapeutic outcomes for patients with ESCC.

## Conclusions

This phase 2 trial demonstrates that neoadjuvant camrelizumab (anti-PD-1) combined with metronomic chemotherapy (IO + MCT) significantly improves pathological responses, achieving a pCR rate of 54.5%, and is associated with a promising 4-year OS rate of 90.9% in locally advanced ESCC, with a manageable toxicity profile. Mechanistically, the therapy reprograms the tumor immune microenvironment by promoting tertiary lymphoid structures and immune infiltration, key factors for therapeutic efficacy. Furthermore, the mitochondrial respiratory chain-derived Immuno-Oncology Related Mitochondrial Complex Signature (IRMCS) was identified as a predictive biomarker, validated in an independent cohort, offering a tool for patient stratification. These findings provide a low-toxicity, high-efficacy neoadjuvant approach and a biomarker-guided strategy for personalized ESCC management, laying the foundation for larger trials to confirm these benefits and integrate IRMCS into clinical decision-making.

## Supplementary Information

Additional file 1: Additional Methods, Figures S1–S8 and Tables S1–S3. Fig S1- [Comprehensive Analysis of Treatment Outcomes]; Fig S2- [DFS and OS outcomes]; Fig S3- [Transcriptomic and pathway enrichment analyses of paired pre- and post-treatment resection specimens of both MCT and IO + MCT groups]; Fig S4- [Changes in the transcriptomic pattern of immune microenvironment before and after treatment in both MCT and IO + MCT groups]; Fig S5- [Bulk RNA-seq analysis and its correlation with pathological response in IO + MCT group]; FigS6- [Validation of CD39 signatures and pathological response in IO + MCT patients by DSP-WTA results]; FigS7- [Correlation between genomic features and pathological responses]; FigS8- [The comprehensive dotchart of pCR rates previously reported in other neoadjuvant treatment regimens for locally advanced resectable ESCC randomized or retrospective clinical studies]; FigS9- [Histopathological identification of tertiary lymphoid structuresin tumor tissue]; Tables S1-[Baseline demographic and clinical characteristics of the included patients]; Tables S2-[Pretreatment clinical stage and posttreatment pathological stage]; Tables S3- [Postoperative complications in patients underwent surgery].

## Data Availability

The transcriptomic data and targeted DNA sequencing data (covering 769 major tumor-associated genes) generated in this study have been deposited in the Genome Sequence Archive (GSA) under BioProject accession number PRJCA056240. Other clinical data from this trial are available from the corresponding author upon reasonable request. Requests should be directed to J. Liu (jieliu@fudan.edu.cn) and must include a detailed research proposal. The request and proposal will be reviewed by the Medical Ethical Committee of Huashan Hospital to assess risks related to patient re-identification. Approved applicants will be required to sign a data access agreement.

## Abbreviations

MCT: Metronomic chemotherapy
ESCC: Esophageal squamous cell carcinoma
DSP-WTA: Digital spatial profiling-whole transcriptomic analysis
mIF: Multiplex immunofluorescent staining
TLS: Tertiary lymphoid structures
pCR: Pathological complete response
OS: Overall survival
ORR: Objective response rates
MPR: Major pathological remissions
ICI: Immune checkpoint inhibitors
TRAEs: Treatment-related adverse effects
MTD: Maximum tolerated dose
DCR: Disease control rates
PFS: Progression-free survival
TRG: Tumor regression grading
FFPE: Formalin-fixed paraffin-embedded
DEGs: Differentially expressed genes
IRMCS: Immuno-oncology related mitochondrial complex signature
TME: Tumor microenvironment
IE: Immune-enriched, non-fibrotic
IE/F: Immune-enriched, fibrotic
F: Fibrotic
D: Desert
WTA: Whole-transcriptome sequencing
ROIs: Regions of interest
PMR: Partial metabolic response
SMD: Stable metabolic disease
CMR: Complete metabolic response
OR: Odds ratio
CI: Confidential interval
SNVs: Single nucleotide variants
CNV: Copy number variation
RNA-seq: Bulk RNA sequencing
DEGs: Differentially expressed genes
GSVA: Gene set variation analysis
EMT: Epithelial-mesenchymal transition
ssGSEA: Single-sample Gene Set Enrichment Analysis
MRC: Mitochondrial respiratory chain
NR: Non-responders
R: Responders
TMB: Tumor mutation burden
MATH: Mutant-allele tumor heterogeneity
OXPHOS: Oxidative phosphorylation

## References

[1] Ajani JA, D’Amico TA, Bentrem DJ, Cooke D, Corvera C, Das P, et al. Esophageal and esophagogastric junction cancers, version 2.2023, NCCN clinical practice guidelines in oncology. J Natl Compr Canc Netw. 2023;21(4):393–422. 37015332 10.6004/jnccn.2023.0019

[2] Wang FH, Zhang XT, Tang L, Wu Q, Cai MY, Li YF, et al. The Chinese Society of Clinical Oncology (CSCO): clinical guidelines for the diagnosis and treatment of gastric cancer, 2023. Cancer Commun. 2024;44(1):127–72. 10.1002/cac2.12516 PMC10794017 38160327

[3] Hoeppner J, Brunner T, Lordick F, Schmoor C, Kulemann B, Neumann UP, et al. Prospective randomized multicenter phase III trial comparing perioperative chemotherapy (FLOT protocol) to neoadjuvant chemoradiation (CROSS protocol) in patients with adenocarcinoma of the esophagus (ESOPEC trial). JCO. 2024;42(17_suppl):LBA1–LBA1. 10.1186/s12885-016-2564-y PMC4952147 27435280

[4] Kojima T, Shah MA, Muro K, Francois E, Adenis A, Hsu CH, et al. Randomized phase III KEYNOTE-181 study of pembrolizumab versus chemotherapy in advanced esophageal cancer. J Clin Oncol. 2020;38(35):4138–48. 33026938 10.1200/JCO.20.01888

[5] Luo H, Lu J, Bai Y, Mao T, Wang J, Fan Q, et al. Effect of camrelizumab vs placebo added to chemotherapy on survival and progression-free survival in patients with advanced or metastatic esophageal squamous cell carcinoma: the ESCORT-1st randomized clinical trial. JAMA. 2021;326(10):916–25. 34519801 10.1001/jama.2021.12836 PMC8441593

[6] Liu J, Li J, Lin W, Shao D, Depypere L, Zhang Z, et al. Neoadjuvant camrelizumab plus chemotherapy for resectable, locally advanced esophageal squamous cell carcinoma (NIC-ESCC2019): a multicenter, phase 2 study. Int J Cancer. 2022;151(1):128–37. 35188268 10.1002/ijc.33976

[7] Liu J, Yang Y, Liu Z, Fu X, Cai X, Li H, et al. Multicenter, single-arm, phase II trial of camrelizumab and chemotherapy as neoadjuvant treatment for locally advanced esophageal squamous cell carcinoma. J Immunother Cancer. 2022;10(3):e004291. 35338088 10.1136/jitc-2021-004291 PMC8961177

[8] Yan X, Duan H, Ni Y, Zhou Y, Wang X, Qi H, et al. Tislelizumab combined with chemotherapy as neoadjuvant therapy for surgically resectable esophageal cancer: a prospective, single-arm, phase II study (TD-NICE). Int J Surg. 2022;103:106680. 35595021 10.1016/j.ijsu.2022.106680

[9] Shang X, Zhao G, Liang F, Zhang C, Zhang W, Liu L, et al. Safety and effectiveness of pembrolizumab combined with paclitaxel and cisplatin as neoadjuvant therapy followed by surgery for locally advanced resectable (stage III) esophageal squamous cell carcinoma: a study protocol for a prospective, single-arm, single-center, open-label, phase-II trial (Keystone-001). Ann Transl Med. 2022;10(4):229. 35280363 10.21037/atm-22-513 PMC8908169

[10] Yang Y, Zhang J, Meng H, Ling X, Wang X, Xin Y, et al. Neoadjuvant camrelizumab combined with paclitaxel and nedaplatin for locally advanced esophageal squamous cell carcinoma: a single-arm phase 2 study (cohort study). Int J Surg. 2024;110(3):1430–40. 38051925 10.1097/JS9.0000000000000978 PMC10942145

[11] Qin J, Xue L, Hao A, Guo X, Jiang T, Ni Y, et al. Neoadjuvant chemotherapy with or without camrelizumab in resectable esophageal squamous cell carcinoma: the randomized phase 3 ESCORT-NEO/NCCES01 trial. Nat Med. 2024;30(9):2549–57. 10.1038/s41591-024-03064-w PMC11405280 38956195

[12] Chen YP, Liu X, Zhou Q, Yang KY, Jin F, Zhu XD, et al. Metronomic capecitabine as adjuvant therapy in locoregionally advanced nasopharyngeal carcinoma: a multicentre, open-label, parallel-group, randomised, controlled, phase 3 trial. Lancet. 2021;398(10297):303–13. 34111416 10.1016/S0140-6736(21)01123-5

[13] Munzone E, Regan MM, Cinieri S, Montagna E, Orlando L, Shi R, et al. Efficacy of metronomic oral vinorelbine, cyclophosphamide, and capecitabine vs weekly intravenous paclitaxel in patients with estrogen receptor-positive, ERBB2-negative metastatic breast cancer: final results from the phase 2 METEORA-II randomized clinical trial. JAMA Oncol. 2023;9(9):1267–72. 37440239 10.1001/jamaoncol.2023.2150 PMC10346502

[14] Basar OY, Mohammed S, Qoronfleh MW, Acar A. Optimizing cancer therapy: a review of the multifaceted effects of metronomic chemotherapy. Front Cell Dev Biol. 2024;12:1369597. 38813084 10.3389/fcell.2024.1369597 PMC11133583

[15] Krajnak S, Battista MJ, Hasenburg A, Schmidt M. Metronomic chemotherapy for metastatic breast cancer. Oncol Res Treat. 2021;45(1–2):12–7. 34794154 10.1159/000520236

[16] Kareva I, Waxman DJ, Lakka KG. Metronomic chemotherapy: an attractive alternative to maximum tolerated dose therapy that can activate anti-tumor immunity and minimize therapeutic resistance. Cancer Lett. 2015;358(2):100–6. 25541061 10.1016/j.canlet.2014.12.039 PMC4666022

[17] Huang J, Xu J, Chen Y, Zhuang W, Zhang Y, Chen Z, et al. Camrelizumab versus investigator’s choice of chemotherapy as second-line therapy for advanced or metastatic oesophageal squamous cell carcinoma (ESCORT): a multicentre, randomised, open-label, phase 3 study. Lancet Oncol. 2020;21(6):832–42. 32416073 10.1016/S1470-2045(20)30110-8

[18] Xu R hua, Luo H, Lu J, Bai Y, Mao T, Wang J, et al. ESCORT-1st: A randomized, double-blind, placebo-controlled, phase 3 trial of camrelizumab plus chemotherapy versus chemotherapy in patients with untreated advanced or metastatic esophageal squamous cell carcinoma (ESCC). J Clin Oncol. 2021. Available from: https://ascopubs.org/doi/10.1200/JCO.2021.39.15_suppl.4000. Cited 2024 Sept 9.

[19] Xing W, Zhao L, Zheng Y, Liu B, Liu X, Li T, et al. The sequence of chemotherapy and toripalimab might influence the efficacy of neoadjuvant chemoimmunotherapy in locally advanced esophageal squamous cell cancer-a phase II study. Front Immunol. 2021;12:772450. 34938292 10.3389/fimmu.2021.772450 PMC8685246

[20] Duan H, Shao C, Pan M, Liu H, Dong X, Zhang Y, et al. Neoadjuvant pembrolizumab and chemotherapy in resectable esophageal cancer: an open-label, single-arm study (PEN-ICE). Front Immunol. 2022;13:849984. 35720388 10.3389/fimmu.2022.849984 PMC9202755

[21] He W, Leng X, Mao T, Luo X, Zhou L, Yan J, et al. Toripalimab plus paclitaxel and carboplatin as neoadjuvant therapy in locally advanced resectable esophageal squamous cell carcinoma. Oncologist. 2022;27(1):e18-28. 35305102 10.1093/oncolo/oyab011 PMC8842349

[22] Lv H, Tian Y, Li J, Huang C, Sun B, Gai C, et al. Neoadjuvant sintilimab plus chemotherapy in resectable locally advanced esophageal squamous cell carcinoma. Front Oncol. 2022;12:864533. 35574384 10.3389/fonc.2022.864533 PMC9098952

[23] Qiao Y, Zhao C, Li X, Zhao J, Huang Q, Ding Z, et al. Efficacy and safety of camrelizumab in combination with neoadjuvant chemotherapy for ESCC and its impact on esophagectomy. Front Immunol. 2022;13:953229. 35911723 10.3389/fimmu.2022.953229 PMC9329664

[24] Karczewski KJ, Francioli LC, Tiao G, Cummings BB, Alföldi J, Wang Q, et al. The mutational constraint spectrum quantified from variation in 141,456 humans. Nature. 2020;581(7809):434–43. 32461654 10.1038/s41586-020-2308-7 PMC7334197

[25] Takahashi T, Kaneoka Y, Maeda A, Takayama Y, Seita K. Neoadjuvant chemotherapy with S-1 plus cisplatin for esophageal squamous cell carcinoma. Update Surg. 2022;74(2):675–83. 10.1007/s13304-021-01167-4 34559400

[26] Yang W, Xing X, Yeung SCJ, Wang S, Chen W, Bao Y, et al. Neoadjuvant programmed cell death 1 blockade combined with chemotherapy for resectable esophageal squamous cell carcinoma. J Immunother Cancer. 2022;10(1):e003497. 35022193 10.1136/jitc-2021-003497 PMC8756283

[27] Chen X, Xu X, Wang D, Liu J, Sun J, Lu M, et al. Neoadjuvant sintilimab and chemotherapy in patients with potentially resectable esophageal squamous cell carcinoma (KEEP-G 03): an open-label, single-arm, phase 2 trial. J Immunother Cancer. 2023;11(2):e005830. 36759013 10.1136/jitc-2022-005830 PMC9923273

[28] Li Y, Zhou A, Liu S, He M, Chen K, Tian Z, et al. Comparing a PD-L1 inhibitor plus chemotherapy to chemotherapy alone in neoadjuvant therapy for locally advanced ESCC: a randomized Phase II clinical trial : a randomized clinical trial of neoadjuvant therapy for ESCC. BMC Med. 2023;21(1):86. 36882775 10.1186/s12916-023-02804-y PMC9993718

[29] Yin J, Yuan J, Li Y, Fang Y, Wang R, Jiao H, et al. Neoadjuvant adebrelimab in locally advanced resectable esophageal squamous cell carcinoma: a phase 1b trial. Nat Med. 2023;29(8):2068–78. 37488287 10.1038/s41591-023-02469-3 PMC10427424

[30] Zhang B, Zhao H, Wu X, Gong L, Yang D, Li X, et al. Perioperative outcomes of neoadjuvant chemotherapy plus camrelizumab compared with chemotherapy alone and chemoradiotherapy for locally advanced esophageal squamous cell cancer. Front Immunol. 2023;14:1066527. 36825006 10.3389/fimmu.2023.1066527 PMC9941171

[31] Zhang G, Yuan J, Pan C, Xu Q, Cui X, Zhang J, et al. Multi-omics analysis uncovers tumor ecosystem dynamics during neoadjuvant toripalimab plus nab-paclitaxel and S-1 for esophageal squamous cell carcinoma: a single-center, open-label, single-arm phase 2 trial. EBioMedicine. 2023;90:104515. 36921563 10.1016/j.ebiom.2023.104515 PMC10024111

[32] Li H, Durbin R. Fast and accurate short read alignment with Burrows-Wheeler transform. Bioinformatics. 2009;25(14):1754–60. 19451168 10.1093/bioinformatics/btp324 PMC2705234

[33] McKenna A, Hanna M, Banks E, Sivachenko A, Cibulskis K, Kernytsky A, et al. The Genome Analysis Toolkit: a mapreduce framework for analyzing next-generation DNA sequencing data. Genome Res. 2010;20(9):1297–303. 20644199 10.1101/gr.107524.110 PMC2928508

[34] Lai Z, Markovets A, Ahdesmaki M, Chapman B, Hofmann O, McEwen R, et al. VarDict: a novel and versatile variant caller for next-generation sequencing in cancer research. Nucleic Acids Res. 2016;44(11):e108. 27060149 10.1093/nar/gkw227 PMC4914105

[35] Wang K, Li M, Hakonarson H. ANNOVAR: functional annotation of genetic variants from high-throughput sequencing data. Nucleic Acids Res. 2010;38(16):e164. 20601685 10.1093/nar/gkq603 PMC2938201

[36] Lek M, Karczewski KJ, Minikel EV, Samocha KE, Banks E, Fennell T, et al. Analysis of protein-coding genetic variation in 60,706 humans. Nature. 2016;536(7616):285–91. 27535533 10.1038/nature19057 PMC5018207

[37] Chen S, Zhou Y, Chen Y, Gu J. fastp: an ultra-fast all-in-one FASTQ preprocessor. Bioinformatics. 2018;34(17):i884-90. 30423086 10.1093/bioinformatics/bty560 PMC6129281

[38] Langmead B, Salzberg SL. Fast gapped-read alignment with Bowtie 2. Nat Methods. 2012;9(4):357–9. 22388286 10.1038/nmeth.1923 PMC3322381

[39] Kim D, Paggi JM, Park C, Bennett C, Salzberg SL. Graph-based genome alignment and genotyping with HISAT2 and HISAT-genotype. Nat Biotechnol. 2019;37(8):907–15. 31375807 10.1038/s41587-019-0201-4 PMC7605509

[40] Love MI, Huber W, Anders S. Moderated estimation of fold change and dispersion for RNA-seq data with DESeq2. Genome Biol. 2014;15(12):550. 25516281 10.1186/s13059-014-0550-8 PMC4302049

[41] Hevler JF, Albanese P, Cabrera-Orefice A, Potter A, Jankevics A, Misic J, et al. MRPS36 provides a structural link in the eukaryotic 2-oxoglutarate dehydrogenase complex. Open Biol. 2023;13(3):220363. 36854377 10.1098/rsob.220363 PMC9974300

[42] Douiev L, Miller C, Keller G, Benyamini H, Abu-Libdeh B, Saada A. Replicative stress coincides with impaired nuclear DNA damage response in COX4-1 deficiency. Int J Mol Sci. 2022;23(8):4149. 35456968 10.3390/ijms23084149 PMC9029573

[43] Cohen B, Altman T, Golani-Armon A, Savulescu AF, Ibraheem A, Mhlanga MM, et al. Co-transport of the nuclear-encoded Cox7c mRNA with mitochondria along axons occurs through a coding-region-dependent mechanism. J Cell Sci. 2022;135(16):jcs259436. 35833493 10.1242/jcs.259436 PMC9481926

[44] Wang C, Lv J, Xue C, Li J, Liu Y, Xu D, et al. Novel role of COX6c in the regulation of oxidative phosphorylation and diseases. Cell Death Discov. 2022;8(1):336. 35879322 10.1038/s41420-022-01130-1 PMC9314418

[45] Liu X, Fu R, Pan Y, Meza-Sosa KF, Zhang Z, Lieberman J. PNPT1 Release from mitochondria during apoptosis triggers decay of poly(A) RNAs. Cell. 2018;174(1):187-201.e12. 29779946 10.1016/j.cell.2018.04.017

[46] Diebold LP, Gil HJ, Gao P, Martinez CA, Weinberg SE, Chandel NS. Mitochondrial complex III is necessary for endothelial cell proliferation during angiogenesis. Nat Metab. 2019;1(1):158–71. 31106291 10.1038/s42255-018-0011-x PMC6521885

[47] Au HC, Seo BB, Matsuno-Yagi A, Yagi T, Scheffler IE. The NDUFA1 gene product (MWFE protein) is essential for activity of complex I in mammalian mitochondria. Proc Natl Acad Sci U S A. 1999;96(8):4354–9. 10200266 10.1073/pnas.96.8.4354 PMC16336

[48] Xu J, Li Y, Fan Q, Shu Y, Yang L, Cui T, et al. Clinical and biomarker analyses of sintilimab versus chemotherapy as second-line therapy for advanced or metastatic esophageal squamous cell carcinoma: a randomized, open-label phase 2 study (ORIENT-2). Nat Commun. 2022;13(1):857. 35165274 10.1038/s41467-022-28408-3 PMC8844279

[49] Bagaev A, Kotlov N, Nomie K, Svekolkin V, Gafurov A, Isaeva O, et al. Conserved pan-cancer microenvironment subtypes predict response to immunotherapy. Cancer Cell. 2021;39(6):845-865.e7. 34019806 10.1016/j.ccell.2021.04.014

[50] Calderaro J, Petitprez F, Becht E, Laurent A, Hirsch TZ, Rousseau B, et al. Intra-tumoral tertiary lymphoid structures are associated with a low risk of early recurrence of hepatocellular carcinoma. J Hepatol. 2019;70(1):58–65. 30213589 10.1016/j.jhep.2018.09.003

[51] Maharjan R, Choi J, Kweon S, Pangeni R, Lee NK, Park SJ, et al. A novel oral metronomic chemotherapy provokes tumor specific immunity resulting in colon cancer eradication in combination with anti-PD-1 therapy. Biomaterials. 2021;281:121334. 34974206 10.1016/j.biomaterials.2021.121334

[52] Bravetti G, Falvo P, Talarico G, Orecchioni S, Bertolini F. Metronomic chemotherapy, dampening of immunosuppressive cells, antigen presenting cell activation, and T cells. A quartet against refractoriness and resistance to checkpoint inhibitors. Cancer Lett. 2023;577(28):216441. 37806515 10.1016/j.canlet.2023.216441

[53] Huang M, O’Shaughnessy J, Zhao J, Haiderali A, Cortés J, Ramsey SD, et al. Association of pathologic complete response with long-term survival outcomes in triple-negative breast cancer: a meta-analysis. Can Res. 2020;80(24):5427–34. 10.1158/0008-5472.CAN-20-1792 32928917

[54] Spring LM, Fell G, Arfe A, Sharma C, Greenup R, Reynolds KL, et al. Pathologic complete response after neoadjuvant chemotherapy and impact on breast cancer recurrence and survival: a comprehensive meta-analysis. Clin Cancer Res. 2020;26(12):2838–48. 32046998 10.1158/1078-0432.CCR-19-3492 PMC7299787

[55] Qin J, Xue L, Hao A, Guo X, Jiang T, Ni Y, et al. Neoadjuvant chemotherapy with or without camrelizumab in resectable esophageal squamous cell carcinoma: the randomized phase 3 ESCORT-NEO/NCCES01 trial. Nat Med. 2024;30(9):2549–57. 38956195 10.1038/s41591-024-03064-w PMC11405280

[56] Guo X, Chen C, Zhao J, Wang C, Mei X, Shen J, et al. Neoadjuvant chemoradiotherapy vs chemoimmunotherapy for esophageal squamous cell carcinoma. JAMA Surg. 2025;160(5):565–74. 40105813 10.1001/jamasurg.2025.0220 PMC11923775

[57] Tang H, Wang H, Fang Y, Zhu JY, Yin J, Shen YX, et al. Neoadjuvant chemoradiotherapy versus neoadjuvant chemotherapy followed by minimally invasive esophagectomy for locally advanced esophageal squamous cell carcinoma: a prospective multicenter randomized clinical trial. Ann Oncol. 2023;34(2):163–72. 36400384 10.1016/j.annonc.2022.10.508

[58] Kato K, Machida R, Ito Y, Daiko H, Ozawa S, Ogata T, et al. Doublet chemotherapy, triplet chemotherapy, or doublet chemotherapy combined with radiotherapy as neoadjuvant treatment for locally advanced oesophageal cancer (JCOG1109 NExT): a randomised, controlled, open-label, phase 3 trial. Lancet. 2024;404(10447):55–66. 38876133 10.1016/S0140-6736(24)00745-1

[59] Yang H, Liu H, Chen Y, Zhu C, Fang W, Yu Z, et al. Neoadjuvant chemoradiotherapy followed by surgery versus surgery alone for locally advanced squamous cell carcinoma of the esophagus (NEOCRTEC5010): a phase III multicenter, randomized, open-label clinical trial. J Clin Oncol. 2018;36(27):2796–803. 30089078 10.1200/JCO.2018.79.1483 PMC6145832

[60] Li C, Zhao S, Zheng Y, Han Y, Chen X, Cheng Z, et al. Preoperative pembrolizumab combined with chemoradiotherapy for oesophageal squamous cell carcinoma (PALACE-1). Eur J Cancer. 2021;144:232–41. 33373868 10.1016/j.ejca.2020.11.039

[61] Ando N, Kato H, Igaki H, Shinoda M, Ozawa S, Shimizu H, et al. A randomized trial comparing postoperative adjuvant chemotherapy with cisplatin and 5-fluorouracil versus preoperative chemotherapy for localized advanced squamous cell carcinoma of the thoracic esophagus (JCOG9907). Ann Surg Oncol. 2012;19(1):68–74. 21879261 10.1245/s10434-011-2049-9

[62] van Hagen P, Hulshof MCCM, van Lanschot JJB, Steyerberg EW, van Berge Henegouwen MI, Wijnhoven BPL, et al. Preoperative chemoradiotherapy for esophageal or junctional cancer. N Engl J Med. 2012;366(22):2074–84. 22646630 10.1056/NEJMoa1112088

[63] Wang H, Tang H, Fang Y, Tan L, Yin J, Shen Y, et al. Morbidity and mortality of patients who underwent minimally invasive esophagectomy after neoadjuvant chemoradiotherapy vs neoadjuvant chemotherapy for locally advanced esophageal squamous cell carcinoma: a randomized clinical trial. JAMA Surg. 2021;156(5):444–51. 33729467 10.1001/jamasurg.2021.0133 PMC7970392

[64] Yang Y, Zhu L, Cheng Y, Liu Z, Cai X, Shao J, et al. Three-arm phase II trial comparing camrelizumab plus chemotherapy versus camrelizumab plus chemoradiation versus chemoradiation as preoperative treatment for locally advanced esophageal squamous cell carcinoma (NICE-2 Study). BMC Cancer. 2022;6(22):506. 10.1186/s12885-022-09573-6 PMC9074348 35524205

[65] Verma V, Shrimali RK, Ahmad S, Dai W, Wang H, Lu S, et al. PD-1 blockade in subprimed CD8 cells induces dysfunctional PD-1+CD38hi cells and anti-PD-1 resistance. Nat Immunol. 2019;20(9):1231–43. 31358999 10.1038/s41590-019-0441-y PMC7472661

[66] Lee M, Heo SH, Song IH, Rajayi H, Park HS, Park IA, et al. Presence of tertiary lymphoid structures determines the level of tumor-infiltrating lymphocytes in primary breast cancer and metastasis. Mod Pathol. 2019;32(1):70–80. 30154578 10.1038/s41379-018-0113-8

[67] Fridman WH, Meylan M, Petitprez F, Sun CM, Italiano A, Sautès-Fridman C. B cells and tertiary lymphoid structures as determinants of tumour immune contexture and clinical outcome. Nat Rev Clin Oncol. 2022;19(7):441–57. 35365796 10.1038/s41571-022-00619-z

[68] Scheper W, Kelderman S, Fanchi LF, Linnemann C, Bendle G, de Rooij MAJ, et al. Low and variable tumor reactivity of the intratumoral TCR repertoire in human cancers. Nat Med. 2019;25(1):89–94. 30510250 10.1038/s41591-018-0266-5

[69] Canale FP, Ramello MC, Núñez N, Araujo Furlan CL, Bossio SN, GorositoSerrán M, et al. CD39 expression defines cell exhaustion in tumor-infiltrating CD8+ T cells. Cancer Res. 2018;78(1):115–28. 29066514 10.1158/0008-5472.CAN-16-2684

[70] Yost KE, Satpathy AT, Wells DK, Qi Y, Wang C, Kageyama R, et al. Clonal replacement of tumor-specific T cells following PD-1 blockade. Nat Med. 2019;25(8):1251–9. 31359002 10.1038/s41591-019-0522-3 PMC6689255

[71] Duhen T, Duhen R, Montler R, Moses J, Moudgil T, de Miranda NF, et al. Co-expression of CD39 and CD103 identifies tumor-reactive CD8 T cells in human solid tumors. Nat Commun. 2018;9(1):2724. 30006565 10.1038/s41467-018-05072-0 PMC6045647

[72] Lin X, Li L, Li S, Li Q, Xie D, Zhou M, et al. Targeting the opening of mitochondrial permeability transition pores potentiates nanoparticle drug delivery and mitigates cancer metastasis. Adv Sci. 2021;8(4):2002834. 10.1002/advs.202002834 PMC7887600 33643797

[73] Vyas S, Zaganjor E, Haigis MC. Mitochondria and cancer. Cell. 2016;166(3):555–66. 27471965 10.1016/j.cell.2016.07.002 PMC5036969

[74] Lord SR, Cheng WC, Liu D, Gaude E, Haider S, Metcalf T, et al. Integrated pharmacodynamic analysis identifies two metabolic adaption pathways to metformin in breast cancer. Cell Metab. 2018;28(5):679-688.e4. 30244975 10.1016/j.cmet.2018.08.021 PMC6224605

[75] Greene J, Segaran A, Lord S. Targeting OXPHOS and the electron transport chain in cancer; molecular and therapeutic implications. Semin Cancer Biol. 2022;1(86):851–9. 10.1016/j.semcancer.2022.02.002 35122973

[76] Skwarski M, McGowan DR, Belcher E, Di Chiara F, Stavroulias D, McCole M, et al. Mitochondrial inhibitor atovaquone increases tumor oxygenation and inhibits hypoxic gene expression in patients with non-small cell lung cancer. Clin Cancer Res. 2021;27(9):2459–69. 33597271 10.1158/1078-0432.CCR-20-4128 PMC7611473

[77] Yan K, Zhang D, Chen Y, Lu W, Huang M, Cai J, et al. Chromosome 11q13 amplification correlates with poor response and prognosis to PD-1 blockade in unresectable hepatocellular carcinoma. Front Immunol. 2023;14:1116057. 37056769 10.3389/fimmu.2023.1116057 PMC10086239

[78] Liu Z, Zhao Y, Kong P, Liu Y, Huang J, Xu E, et al. Integrated multi-omics profiling yields a clinically relevant molecular classification for esophageal squamous cell carcinoma. Cancer Cell. 2023;41(1):181-195.e9. 36584672 10.1016/j.ccell.2022.12.004

