# Supplementary material for: Neoadjuvant camrelizumab combined with metronomic chemotherapy in patients with advanced esophageal squamous cell carcinoma: a pilot randomized phase 2 trial
Source: BMC Med. 2026 Mar 3;24:205. doi: 10.1186/s12916-026-04758-3 (PMC13063907; doi:10.1186/s12916-026-04758-3)
Supplement: Supplementary file 1 — Additional file 1: Additional Methods, Figures S1–S8 and Tables S1–S3. Fig S1- [Comprehensive Analysis of Treatment Outcomes]; Fig S2- [DFS and OS outcomes]; Fig S3- [Transcriptomic and pathway enrichment analyses of paired pre- and post-treatment resection specimens of both MCT and IO + MCT groups]; Fig S4- [Changes in the transcriptomic pattern of immune microenvironment before and after treatment in both MCT and IO + MCT groups]; Fig S5- [Bulk RNA-seq analysis and its correlation with pathological response in IO + MCT group]; FigS6- [Validation of CD39 signatures and pathological response in IO + MCT patients by DSP-WTA results]; FigS7- [Correlation between genomic features and pathological responses]; FigS8- [The comprehensive dotchart of pCR rates previously reported in other neoadjuvant treatment regimens for locally advanced resectable ESCC randomized or retrospective clinical studies]; FigS9- [Histopathological identification of tertiary lymphoid structuresin tumor tissue]; Tables S1-[Baseline demographic and clinical characteristics of the included patients]; Tables S2-[Pretreatment clinical stage and posttreatment pathological stage]; Tables S3- [Postoperative complications in patients underwent surgery]. [file 12916_2026_4758_MOESM1_ESM.docx]

Additional Methods

Multiplex immunofluorescent staining and analysis

Multiplex immunofluorescence (mIF) assay was conducted to visualize multiple immune components, including T lymphocytes (CD4, CD8), Macrophages (CD68, CD163), tumor epithelial cells (PanCK), and inhibitory markers (CD39, PD-1, and PD-L1), in formalin-fixed paraffin-embedded (FFPE) tumor slides. Four-μm thick slides were prepared from the FFPE ESCC tissues, underwent deparaffinized, rehydrated, washed, and epitope retrieval through boiling in Tris-EDTA buffer (pH=9; Klinipath #643901, the Netherlands). Endogenous peroxidase was blocked by incubation in Antibody Block/Diluent (PerkinElmer #72424205, USA), and the slides were then blocked in 0.05% Tween solution. Primary and secondary antibody incubation was performed with Opal Polymer HRP Ms+Rb (2414515, PerkinElmer, Massachusetts, USA) followed by TSA visualization with the Opal seven-color IHC Kit (NEL797B001KT, PerkinElmer) and TSA Coumarin system (NEL703001KT, PerkinElmer). Primary antibodies for CD8 (ZSGB-Bio Cat# ZA-0508, RRID:AB_2890107, 1:100), CD4 ((ZSGB-Bio Cat# ZM-0418, RRID:AB_2890106, 1:100), CD39 (Abcam Cat# ab223842, RRID:AB_2889212, 1:1000), CD68 (ZSGB-Bio Cat# ZM-0060, RRID:AB_2904190, 1:500), CD163 (Abcam Cat# ab189915, RRID:AB_3493124, 1:100), PanCK (ZSGB-Bio Cat# ZM-0069, RRID:AB_2941997, 1:200), PD-1 (ZSGB-Bio Cat# ZM-0381, RRID:AB_2921363, 1:50) and PD-L1 (Abcam Cat# ab228462, RRID:AB_2827816 1:25) were incubated overnight at 4℃. Finally, DAPI was applied for counterstaining, and slides were mounted using glycerine. The PerkinElmer Vectra (Vectra 3.0.5) was used to scan the slides, and the InForm Advanced Image Analysis software (inForm 2.3.0; PerkinElmer) was used for analysis. Density calculated by the counts of stained cells in the corresponding area was used for analyses.

DNA Extraction and Targeted Sequencing

Genomic DNA was isolated from the formalin-fixed paraffin-embedded (FFPE) tissue specimens and matched peripheral blood lymphocytes using the black PREP FFPE DNA Kit (Analytik Jena, Germany) and Tiangen Whole Blood DNA Kits (Tiangen, Beijing, PRC) according to the manufacturer’s instructions. Next, genomic DNA was sheared into 150-200 bp fragments for sequencing with a Covaris E220evolution Focused ultrasonicator (RRID:SCR_019817) after quantified by a Qubit dsDNA HS Assay kit (Life Technologies, USA). Fragmented DNA libraries were constructed using a KAPA HTP Library Preparation Kit (KAPA Biosystems, Massachusetts, USA) according to the manufacturer’s instructions. A custom capture panel (Genecast, Beijing, China) with 769 major tumor-associated genes was applied to capture the DNA libraries. The captured DNA fragments were then subjected to Novaseq 6000 (RRID:SCR_016387) processing for paired-end sequencing.

Single Nucleotide Variants (SNVs) Calling

The raw data without low-quality reads were aligned to the human reference genome (Hg19, NCBI Build 37.5) using the Burrows-Wheeler Aligne(40). Then, the Picard (RRID:SCR_006525) and Computational Genomics Analysis Tools (RRID:SCR_006390) (41) were utilized for making duplicates and realignment, respectively. After that, VarDict (version 1.5.1, RRID:SCR_023658)(42) was employed to call somatic SNVs while FreeBayes (RRID:SCR_010761) was performed to merge compound heterozygous mutations, and then ANNOVAR (RRID:SCR_012821) was applied to annotate the mutations. Paired genomic DNA samples were used as a control to distinguish somatic mutations from inherited germline variations(43). The calling results were subsequently filtered with custom and more stringent criteria as follows: mutant allele frequency ≥5%; mutant allele support reads ≥5; not located in intergenic regions or intronic regions and not synonymous SNVs; allele frequency ≤0.2% in the ExAC database (44) and Genome Aggregation Database(45).

Copy Number Variation (CNV) calling

All blood cell samples obtained from patients were used to construct a copy number baseline for negative control and the CNV from FFPE tissue samples was called for each patient using a CNV kit, V0.9.2. The thresholds of copy numbers ≥3 and ≤1.2 were employed to categorize altered regions into CNV gains (amplifications) and copy number losses (deletions).

Bulk RNA-seq

Total RNA was isolated from paraffin-embedded tissue using a RNeasy Plus Mini kit (Qiagen). The concentration and integrity of total RNA were then evaluated using a Qubit RNA HR Assay Kit (Thermo Fisher Scientific) and 2100 Bioanalyzer (Agilent, RRID:SCR_018043). RNA sample libraries were constructed using a TruSeq Stranded mRNA Prep kit (Illumina) in accordance with the manufacture’s manual. After enrichment and purification, libraries were sequenced on a Illumina NovaSeq 6000 Sequencing System (RRID:SCR_016387) with 150 bp paired-end reads. The obtained raw paired-end reads were trimmed using the fastp(46) and bowtie2 (47) tool to remove low-quality reads, adapter contaminants and rRNA data. Then, raw read data were aligned to the human reference genome (build 37.2) using the hisat2 (version 2.1.0, RRID:SCR_015530)(48) tool. DESeq2 (RRID:SCR_015687) (49) and the upper quartile normalization method were used to normalize the raw read counts.

Differentially expressed genes and pathway enrichment analysis

Differentially expressed genes (DEGs) between comparisons were analyzed using Deseq2 v1.30 fold change ≥2 and P-values ≤0.05 were considered the cutoff criteria for DEGs analysis. Gene ontology (GO) and Reactom pathway enrichment were analyzed by ClusterProfiler v.4.4.4 (RRID:SCR_016884) using DEGs. The hallmark score was calculated using Gene Sets Variation Analysis (GSVA, v.1.46, RRID:SCR_021058) and msigdbr (v7.5.1, RRID:SCR_022870). Hallmark pathways were considered significant at P-values ≤0.05. Gene signatures of significant pathways were used. Briefly, for each tumor sample and hallmark score, we obtained a score between [-2, 2], with extreme values close to 2 or -2, indicating the extent of enrichment of gene signatures.

Immuno-oncology related mitochondrial complex signature

Seven DEGs, including *MRPS36* (50), *COX4I* (51), *COX7C* (52), *COX6C* (53), *PNPT1* (54), *UQCRQ* (55), and *NDUFA1* (56) which belong to mitochondrial respiratory chain and oxidative phosphorylation as previous studies reported, were selected as the immuno-oncology related mitochondrial complex signature (IRMCS). IRMCS score was calculated for each sample using GSVA (v.1.46, RRID:SCR_021058) with single-sample GSEA (ssGSEA, RRID:SCR_003199) as an enrichment method. ORIENT-2 immunotherapy cohort(57) for advanced ESCC patients treated with immunotherapy alone was used to verify whether the IRMCS could be widely applied in ESCC.

Evaluation of TME-related signature and cell scores

The abundance of 28 major cell types of infiltration was calculated using GSVA (v.1.46, RRID:SCR_021058) with the ssGSEA (RRID:SCR_003199) algorithm based on RNA-seq expression profiles. ESTIMATE v.1.0.13 (RRID:SCR_026090) was utilized to calculate the immune score and stromal scores using bulk RNA-seq data. To determine the correlation between the therapeutic regimen associated with inflamed TME and clinical benefits, we further explored the dynamic changing of the TME subtypes including Immune-enriched, non-fibrotic (IE), Immune-enriched, fibrotic (IE/F), Fibrotic (F), Desert (D) by referring to the previous study(58). RNA-seq data from our study were classified by a KNN model using an R package CLASS.

Identification of TLS

Tertiary lymphoid structures (TLS) were identified and quantified using a multi-modal approach to ensure rigorous morphological and architectural validation (59). Based on established criteria, TLS were further classified as: i) Primary follicles (FL-I): defined as organized aggregates of B cells and T cells lacking a discernible germinal center; and ii) Secondary follicles (FL-II): defined as lymphoid aggregates containing a clearly formed germinal center. As shown in the immunofluorescence plot in Additional file 1: Fig. S9, red fluorescence in the circles marked secondary lymphoid follicles dominated by CD20, yellow fluorescence marked CD3 surrounding lymphoid follicles, and esophageal squamous epithelium was marked with green fluorescence in the boxes. HE shows mature tertiary lymphoid structures with germinal centers, and highly endothelial veins are indicated by arrows. All intratumoral TLS were independently quantified by two board-certified pathologists who were blinded to all clinical and treatment data, and the average count was used for subsequent analysis.

Whole-transcriptome sequencing (WTA) and GeoMX DSP

After conventional deparaffinization and rehydration, FFPE tumor sections were hybridized with probes in the Cancer Transcriptome Altas panel (NanoString, Seatle, WA) at 37℃ overnight and incubated with fluorescent antibodies including anti-CD3 (Roche Cat# 05278422001, RRID:AB_2335978), anti-CD20 (ABclonal, Wuhan, China), anti-Pan-CK (NanoString) to facilitate the identification of tissue morphology. Based on the fluorescent markers and consecutive HE staining slides, the spatially resolved ROIs were selected including the pan-CK+/- tumor areas, the whole TLSs zones indicated by CD3+ and CD20+ cell-clustered regions, and the segmented TLSs of separately T cell zones (the CD3+ cell-clustered regions in the outer layer of TLSs) and B cell zones (the CD20+ and CD3- cell-clustered regions in the center of TLSs). Conjugated target-specific oligos of each ROI were collected in 96-well plates after ultraviolet irradiation. Library preparation was performed according to the manufacturer’s instructions (NanoString GeoMx Tools (RRID:SCR_023424)) and sequenced on a NextSeq 550 (Illumina, San Diego, CA, RRID:SCR_016381). After quality check, raw counts were normalized with the Q3 normalization method, which was further used for quantification of cell populations in each ROI using SpatialDecon with the reference safe TME matrix. Clean counts were utilized to assess disparities between groups. Heatmaps were drawn using the Complex Heatmap package (v2.8.0) of R (v4.1.0).

Statistical analysis

All statistical analyses were conducted using R (v4.2.2). The boxplots were constructed with the center line and box boundaries representing the median, 25th and 75th percentiles, while the upper and lower whiskers illustrated 75th percentiles +1.5× interquartile range and 25th percentiles −1.5× interquartile range, respectively. In addition, points were used to denote outliers. A non-parametric two-sided Wilcoxon rank-sum test was employed to compare two populations, and if the comparisons were paired resection specimens, a two-sided paired Wilcoxon rank-sum test was used. The frequencies of genomic alterations were compared using Fisher's exact test. The progression-free survival rate was calculated via the Kaplan-Meier method with the Log-rank test using survminer (v.0.4.9, RRID:SCR_021094). A significance level of *P*-value is less than 0.05 was used to determine statistical significance.

Additional Figures:


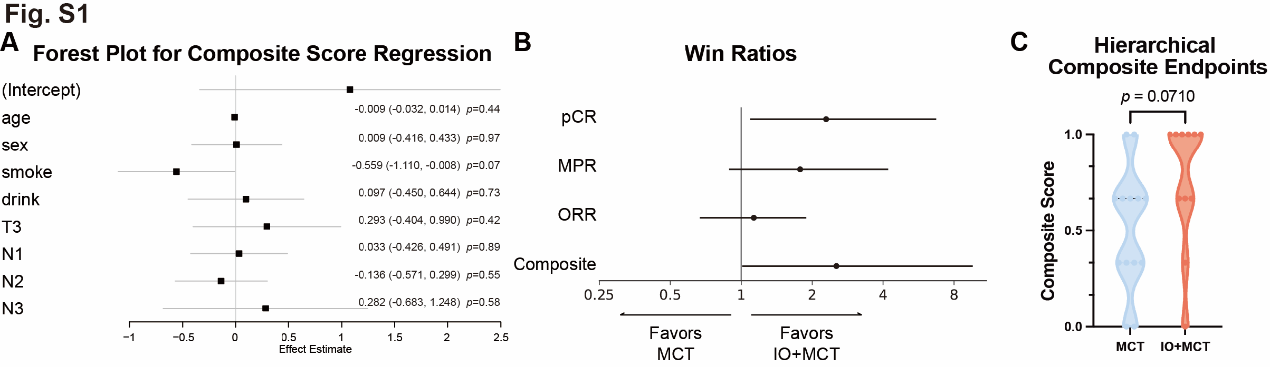


**Fig. S1** Comprehensive Analysis of Treatment Outcomes: Regression, Win Ratio, and Hierarchical Composite Endpoints Methods **A.** Forest Plot of Regression Analysis for Composite Score: Associations between treatment outcomes in both groups and factors including age, gender, cigarette-smoking history, alcohol-drinking history, TNM stage and tumor location**. B.** The Win Ratio method was employed to compare the primary endpoints (including pCR, MPR, and ORR) between the two groups. **C.** Hierarchical Composite Endpoints method was employed to compare the primary endpoints (including pCR, MPR, and ORR) between the two groups.
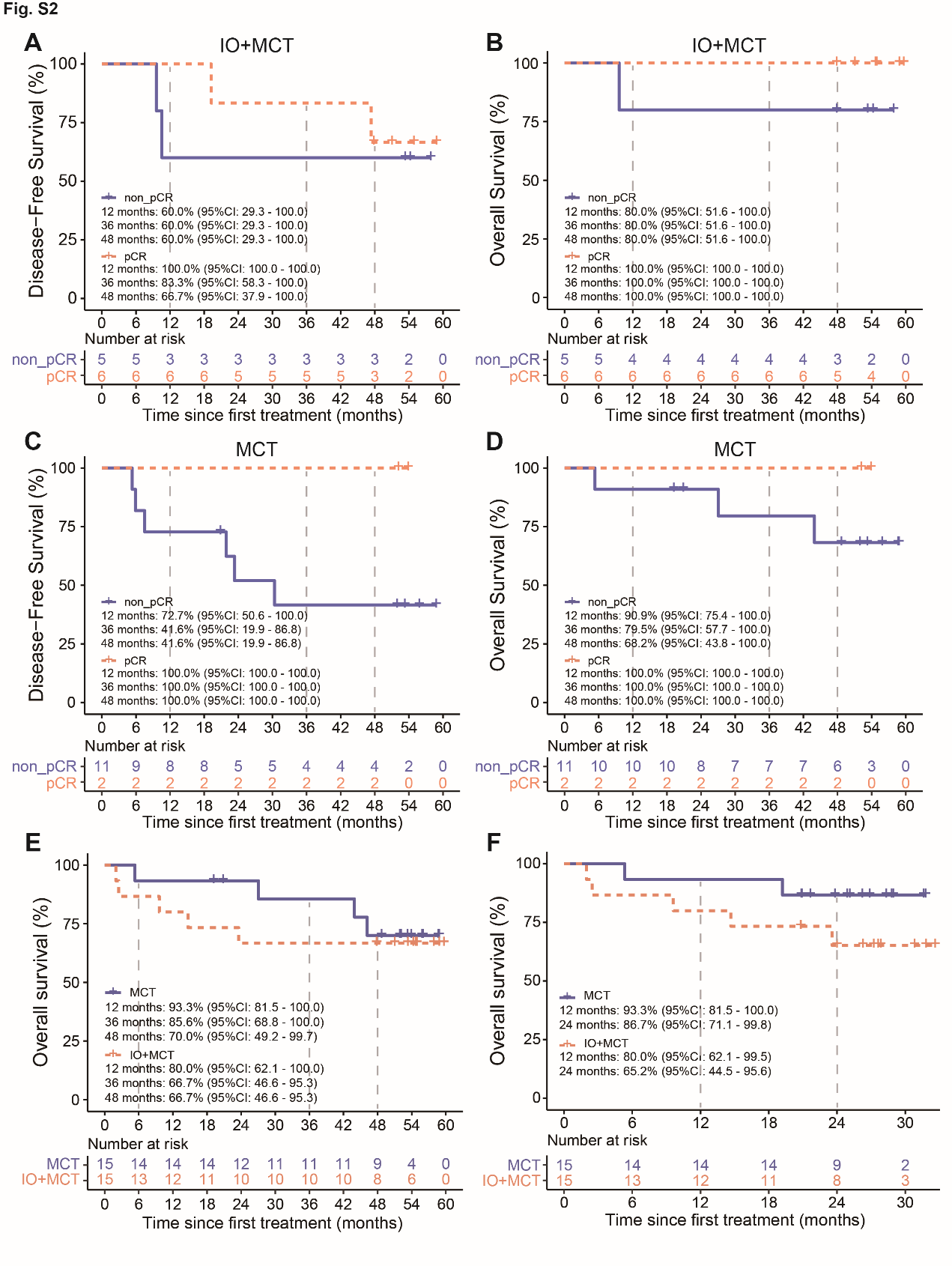


**Fig. S2** DFS and OS outcomes.

**A-B.** Comparison of 4-year DFS **(A)** and OS **(B)** between patients with pCR and those without pCR in the IO+MCT group. **C-D.** Comparison of 4-year DFS **(C)** and OS **(D)** between patients with pCR and those without pCR in the MCT group. **E-F.** ITT analysis for Kaplan-Meier curves of the 4-year overall survival rate (E) and 2-year overall survival rate (F) for patients in MCT and IO+MCT groups.


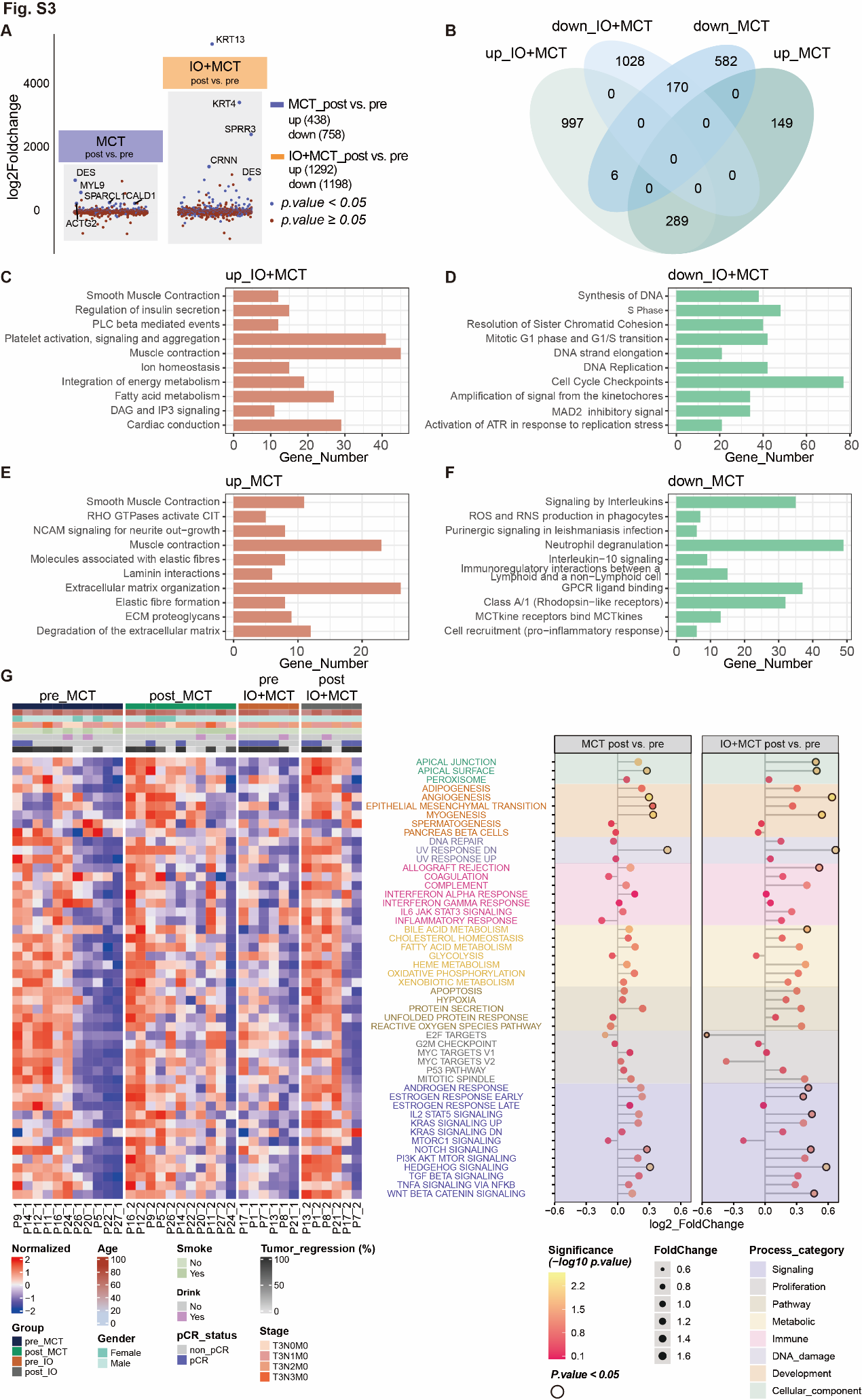
 **Fig. S3** Transcriptomic and pathway enrichment analyses of paired pre- and post-treatment resection specimens of both MCT and IO+MCT groups. **A.** DEGs identified in paired pre- and post-treatment samples. **B.** Paired DEGs analysis of pre- and post-treatment samples for individual patients, showing distinct transcriptomic changes induced by IO+MCT versus MCT treatments. **C-F.** Pathway enrichment analysis for up-and down-regulated DEGs between pre- and post-treatment samples in IO+MCT group (C-D) and MCT group (E-F). **G.** GSVA of hallmark gene sets in paired pre- and post-treatment samples of both MCT and IO+MCT groups. Statistical analysis: (G) paired t-test.

**
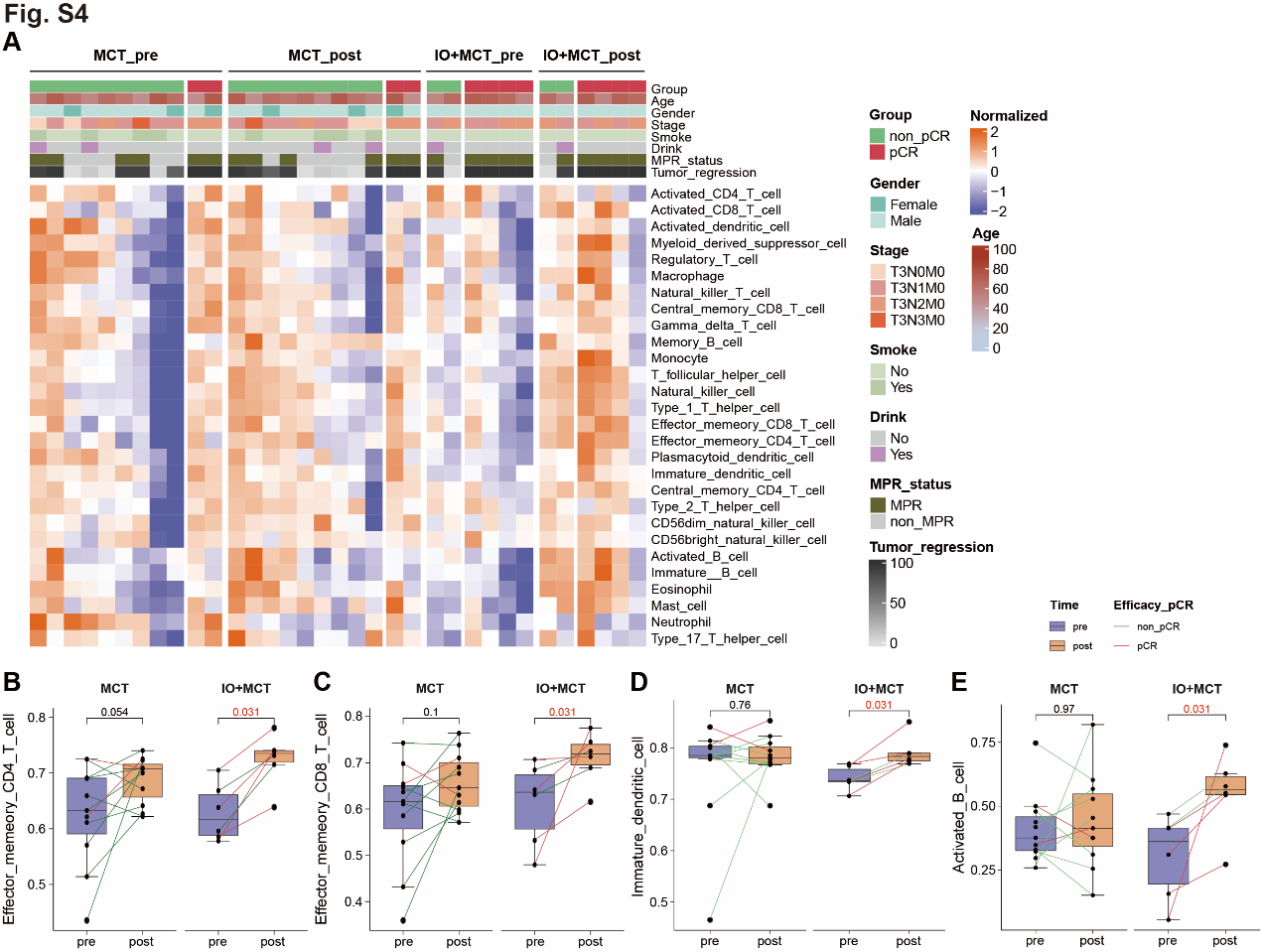
**

**Fig. S4** Changes in the transcriptomic pattern of immune microenvironment before and after treatment in both MCT and IO+MCT groups.

**A.** The transcriptomic pattern of immune cells in the paired pre- and post-treatment samples in both groups. **B-E.** Changes in the abundance of effector memory CD4+ T cells(B), effector memory CD8+ T cells(C), immature dendritic cells(D) and activated B cells(E) treated with IO+ MCT or with MCT only between pre- and post-treatment. **F.** The number of primary and secondary follicle-like TLSs were counted for analyses based on the staining of pathological tissue sections of surgical resections of non-pCR and pCR patients. Statistical analysis: (B-E) paired t-test; (F) independent t-test.


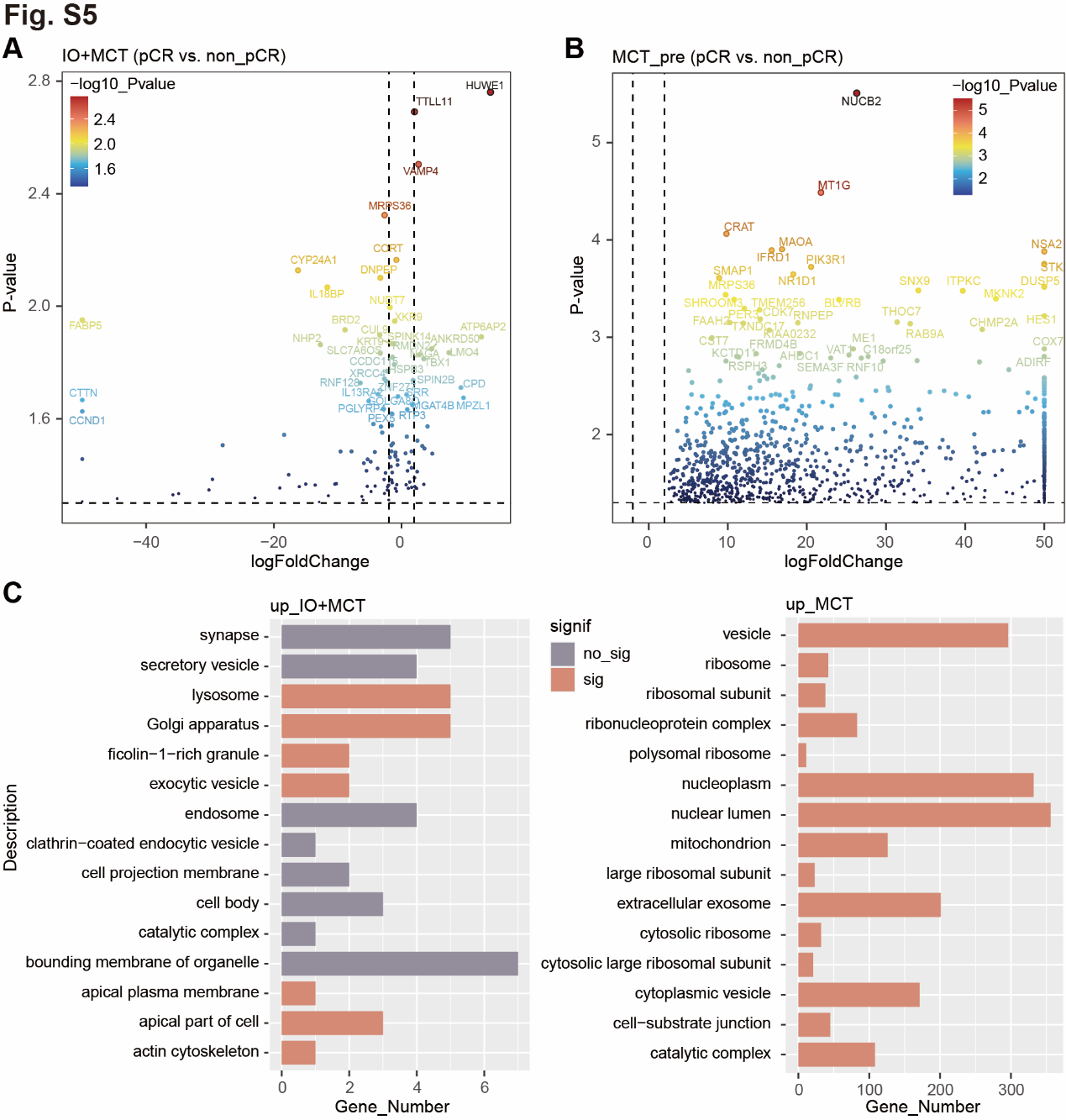


**Fig. S5** Bulk RNA-seq analysis and its correlation with pathological response in IO+MCT group. **A-B.** DEGs between pre-treatment samples of IO+MCT patients with pCR and non-pCR. **A.** A total of 571 DEGs were identified in the IO+MCT group. **B.** A total of 1,267 DEGs were identified in the MCT group. **C.** Functional enrichment analyses of up-regulated genes in the IO+MCT and MCT Group.


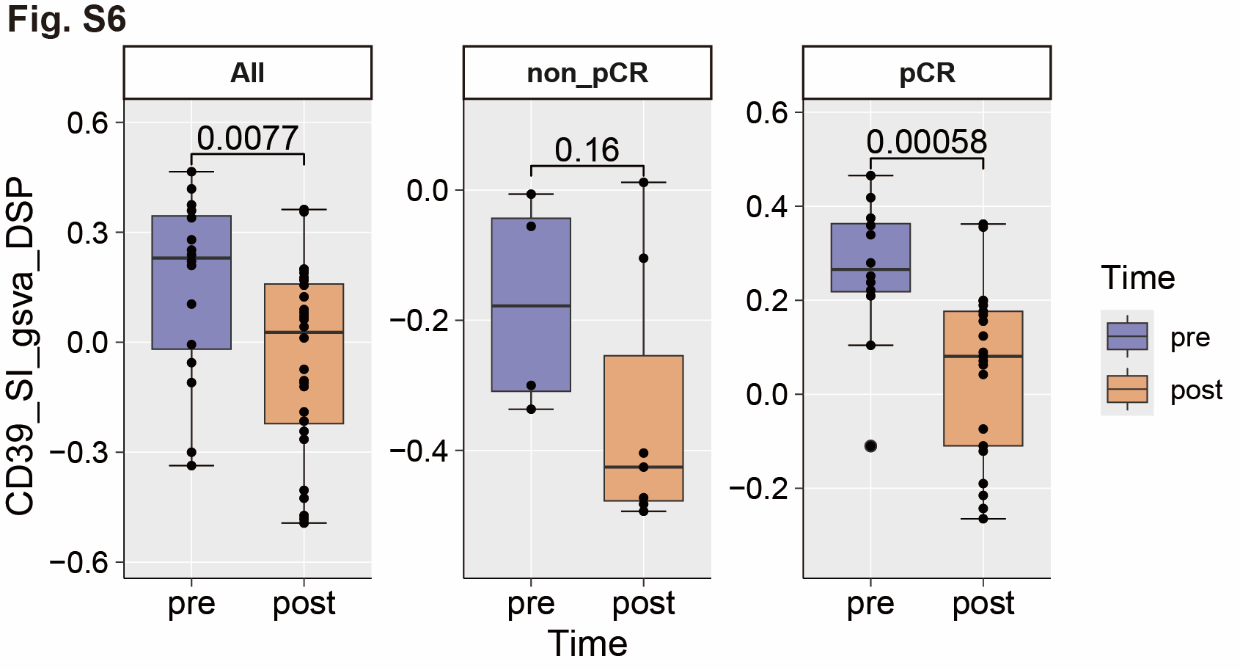


**Fig. S6** Validation of CD39 signatures and pathological response in IO+MCT patients by DSP-WTA results. A-B. Changes of CD39 signatures between pre- and post- IO+MCT treatment in all(A), pCR(B) and non-pCR(C) patients based on the DSP-WTA results. *P* value was calculated with paired two-sided Wilcoxon rank-sum test.


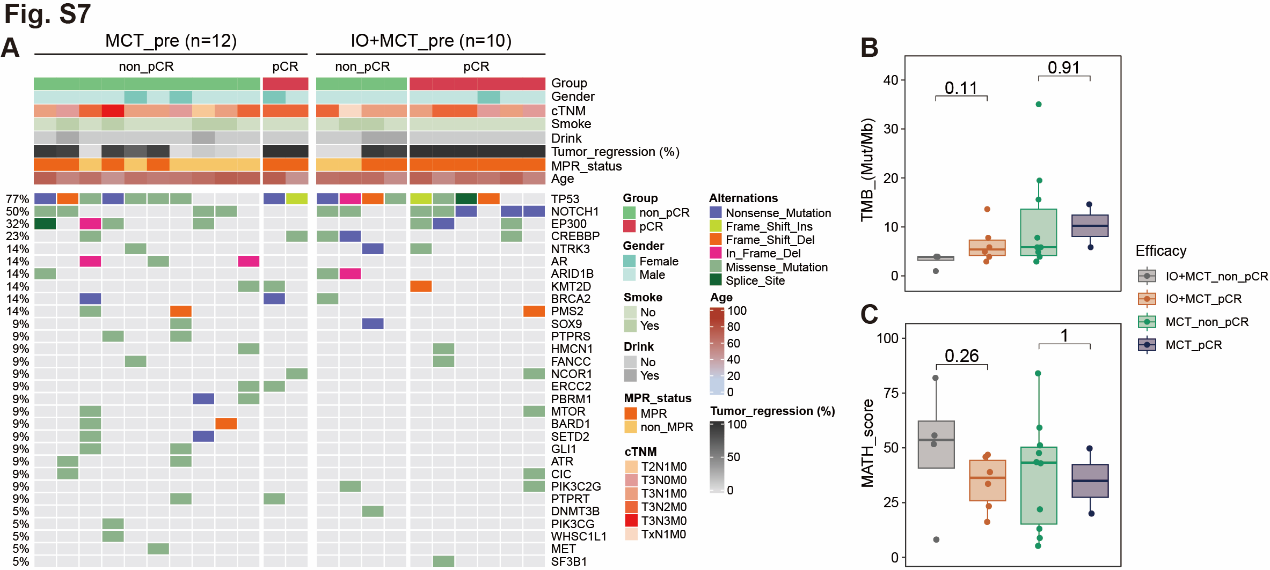


**Fig. S7** Correlation between genomic features and pathological responses. **A.** Mutation frequency and alteration types of the most commonly mutated genes in ESCC patients, based on targeted DNA sequencing of pre-treatment biopsies. **B-C.** Comparison of TMB (B) and MATH scores (C) between patients with pCR and non-pCR in the IO+MCT and MCT groups. *P* value was calculated using a non-parametric two-sided Wilcoxon rank-sum test for comparison of the TMB and MATH of the patients with pCR and non-pCR in the IO+MCT and MCT groups.


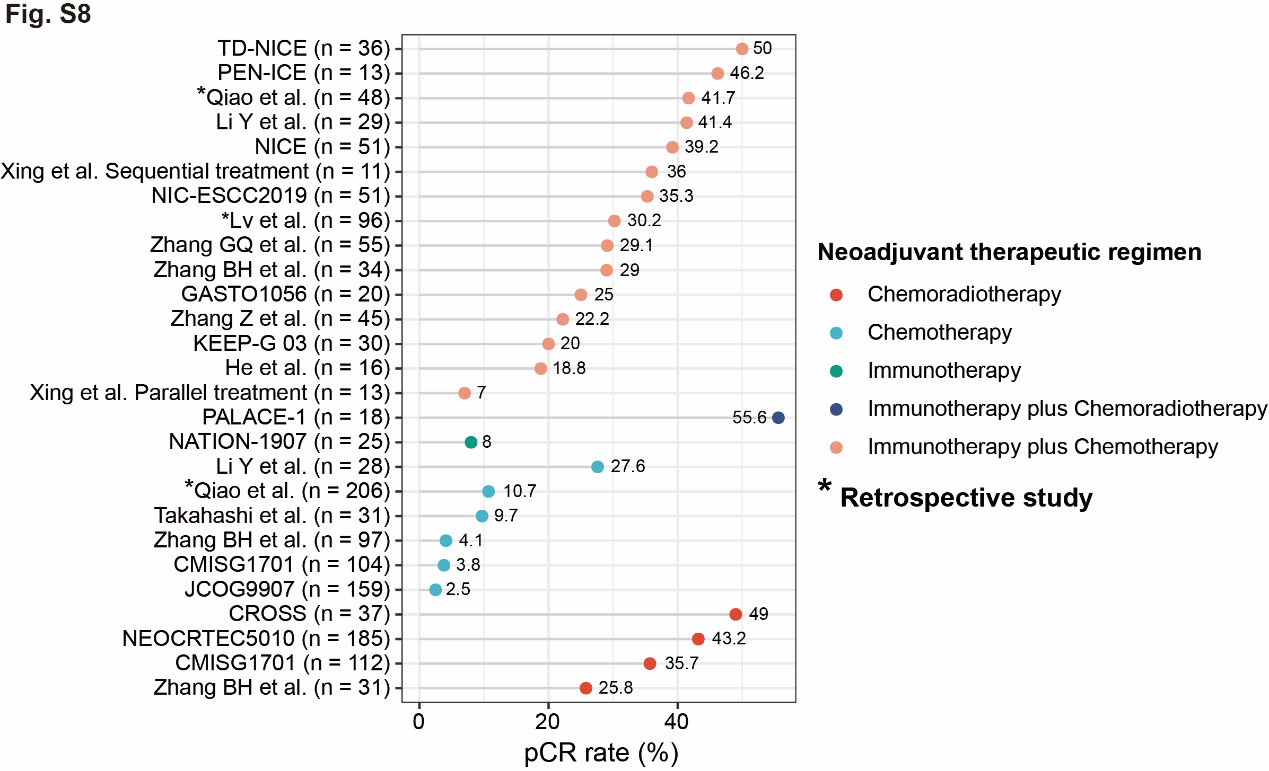


**Fig. S8** The comprehensive dotchart of pCR rates previously reported in other neoadjuvant treatment regimens for locally advanced resectable ESCC randomized or retrospective clinical studies.


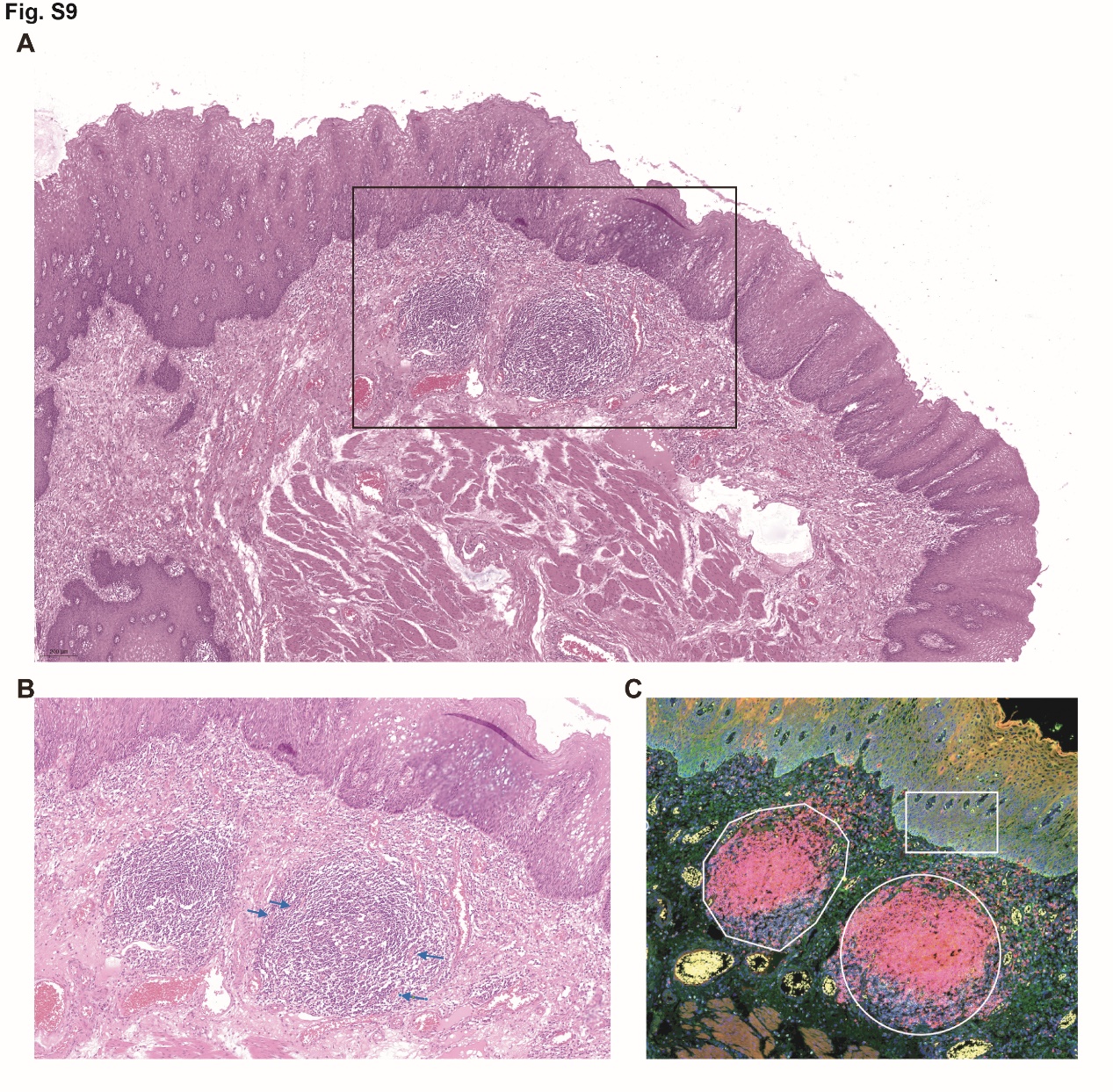


**Fig. S9** Histopathological identification of TLS in tumor tissue. **A.** HE staining showing lymphocyte aggregates (50× magnification). **B.** HE staining demonstrates a mature tertiary lymphoid structure with a visible germinal center; arrows indicate high endothelial venules (100× magnification). **C.** Representative immunofluorescence image of a serial tissue section. Within the circled region, a secondary lymphoid follicle is predominantly composed of CD20+ B cells (red). The surrounding T-cell zone shows CD3+ T cells (yellow). Boxed area highlights the adjacent esophageal squamous epithelium (green autofluorescence).

**Additional Tables:**

**Table S1. Baseline demographic and clinical characteristics of the included patients**

|  | **Camrelizumab plus MCT**  **(*n* = 11)** | **MCT**  **(*n* = 13)** | **P value** |
| --- | --- | --- | --- |
| **Age, years** | 65 (58-67) | 63 (54-71) | 0.286 |
| **Sex** |  |  |  |
| Female | 1(9.1%) | 3 (23.1%) | 0.36 |
| Male | 10(90.9%) | 10(76.9%) |  |
| **ECOG performance status**^A^ |  |  |  |
| 0 | 11 (100%) | 13 (100%) |  |
| 1 and 2 | 0 | 0 |  |
| **Cigarette-smoking history** |  |  |  |
| Yes | 2 (18.2%) | 5 (38.5%) | 0.276 |
| No | 9 (81.8%) | 8 (61.5%) |  |
| **Alcohol-drinking history** |  |  |  |
| Yes | 2 (18.2%) | 3 (23.1%) | 0.769 |
| No | 9 (81.8%) | 10 (76.9%) |  |
| **Tumor size stage** |  |  |  |
| T2 | 1 (9.1%) | 1（7.7%） | 0.902 |
| T3 | 10 (90.9%) | 12（92.3%） |  |
| **Nodal stage** |  |  |  |
| N0 | 3 (27.3%) | 2 (15.4%) | 0.735 |
| N1 | 5 (45.5%) | 6 (46.2%) |  |
| N2 | 3 (27.3%) | 4 (30.8%) |  |
| N3 | 0 (0%) | 1 (7.7%) |  |
| **Tumor location** |  |  |  |
| Upper | 3 (27.3%) | 0(0%) | 0.23 |
| Middle | 4 (36.4%) | 8 (61.5%) |  |
| Lower | 4 (36.4%) | 5 (38.5%) |  |
| **Stage** |  |  |  |
| II | 3 | 2 | 0.531 |
| III | 8 | 10 |  |
| IVA | 0 | 1 |  |

Data are n (%) or median (IQR). ^A^ECOG=Eastern Cooperative Oncology Group.

The Chi-square test was used to assess differences between the two groups. p < 0.05 was considered statistically significant.

**Table S2. Pretreatment clinical stage and posttreatment pathological stage**


**Table S3. Postoperative complications in patients underwent surgery**
